# Intrapopulation Genome Size Variation in *D. melanogaster* Reflects Life History Variation and Plasticity

**DOI:** 10.1371/journal.pgen.1004522

**Published:** 2014-07-24

**Authors:** Lisa L. Ellis, Wen Huang, Andrew M. Quinn, Astha Ahuja, Ben Alfrejd, Francisco E. Gomez, Carl E. Hjelmen, Kristi L. Moore, Trudy F. C. Mackay, J. Spencer Johnston, Aaron M. Tarone

**Affiliations:** 1 Department of Entomology, Texas A&M University, College Station, Texas, United States of America; 2 Department of Biological Sciences, North Carolina State University, Raleigh, North Carolina, United States of America; 3 University of Texas Health Science Center, Houston, Texas, United States of America; 4 Baylor Human Genome Sequencing Center, Houston, Texas, United States of America; 5 Department of Soil and Crop Science, Texas A&M University, College Station, Texas, United States of America; The University of North Carolina at Chapel Hill, United States of America

## Abstract

We determined female genome sizes using flow cytometry for 211 *Drosophila melanogaster* sequenced inbred strains from the *Drosophila* Genetic Reference Panel, and found significant conspecific and intrapopulation variation in genome size. We also compared several life history traits for 25 lines with large and 25 lines with small genomes in three thermal environments, and found that genome size as well as genome size by temperature interactions significantly correlated with survival to pupation and adulthood, time to pupation, female pupal mass, and female eclosion rates. Genome size accounted for up to 23% of the variation in developmental phenotypes, but the contribution of genome size to variation in life history traits was plastic and varied according to the thermal environment. Expression data implicate differences in metabolism that correspond to genome size variation. These results indicate that significant genome size variation exists within *D. melanogaster* and this variation may impact the evolutionary ecology of the species. Genome size variation accounts for a significant portion of life history variation in an environmentally dependent manner, suggesting that potential fitness effects associated with genome size variation also depend on environmental conditions.

## Author Summary

Genome size evolution is ubiquitous, and–even after decades of research–mysterious. There are two major classes of hypotheses regarding genome size evolution, those that attribute its causes to evolutionarily neutral processes and those that suggest a role for selection. Numerous correlations between genome size and fitness-related phenotypes have been documented, suggesting selection could play a role in genome size evolution. Unfortunately, many of the effects in those studies are confounded with factors that could provide alternative explanations. Here, we show that 211 inbred strains of *Drosophila melanogaster* exhibit abundant variation in genome size, which correlates with life history traits in a temperature-dependent manner. Gene expression analyses suggest a role for differences in metabolism between strains with large and small genomes. Thus, there is genetic variation in genome size within *D. melanogaster*, and this variation is connected to variation in environmentally dependent life history traits. These observations indicate that selection is indeed a potential mechanism by which genome size can evolve. Our results also suggest that higher levels of genetic architecture may explain some of the genetic contribution to biologically important complex traits and raise the possibility that nucleotide quantity can contribute to phenotype in addition to quality.

## Introduction

Genome size evolution is extensive and ubiquitous. However, the mechanisms by which this occurs are poorly understood and hotly debated, despite a wealth of information connecting genome size shifts to numerous phenotypes, lineages, and abiotic environments [1]–[16]. One critical component of this debate is whether selection can act on genome size, or if it is a neutrally evolving cellular character. Proponents of genome size evolution point to the association of genome size with cell size and rate of cell division, which impact phenotypes important to fitness [10], [11], [16]–[19]. For instance, it has been suggested that *Drosophila* species with longer development times tend to have larger genomes [10]. Similarly, “weedy” plant species have been hypothesized to have smaller genomes and short generation times [18]. Recently, a review of the genome size literature in numerous endothermic and ectothermic species has made a strong case that genome size evolution could play a role in temperature-size interactions, which could potentially explain adaptive variation in numerous species [16]. While the connection between genome size variation and phenotype is generally recognized for order of magnitude changes in genome size and for interspecific phenotype comparisons, there is little evidence that these effects act on the relatively small magnitude of variation in genome size expected within a species – especially in non-plants. Alternatively, neutral factors such as founder effects and random drift [15], [19] and changes in insertion/deletion balance [13] have been proposed as mechanisms for intraspecific changes in genome size. Consequently, conflicting theories of genome size evolution exist and neither camp has definitively documented the potential for selection rather than chance as a driving force.

In order for selection to drive genome size evolution (either directly or indirectly), variation in genome size must occur within a species and be connected to a phenotype that impacts fitness. It is for this reason that repeated attempts have been made to observe conspecific variation in genome size [1]–[5]. Often they have been linked to phenotypic analyses in wild individuals. However, because the observed phenotypic variation is confounded with environmental variation and because it is difficult to achieve high levels of independently replicated genome size and phenotypic measures from wild populations; it has been difficult to develop a compelling case that genome size is associated with variation in fitness related traits. In addition, some of these studies may also be affected by environmental interactions with genome size measurement technology (e.g. anthocyanin in plants can bias genome size measures [20]). Accordingly, studies of wild individuals have not resolved this debate.

One of the more compelling selection-based arguments in the recent genome size literature has linked nucleotypic effects (genome size is connected with replication rate and cell size) of genome size variation to thermal responses [16]. Many ectotherms follow the temperature-size rule, where body size increases as temperature decreases [21]. *Drosophila* species follow this rule [22] and also demonstrate population-level differences in size (across continents) that mirror this pattern, where strains derived from cooler environments are also larger than those from warmer environments [23]–[27]. Since this pattern has appeared on numerous continents, it is clear that larger size at higher latitudes/cooler temperatures is adaptive for *Drosophila* species. *Drosophila* body size is also correlated with numerous other life history traits in a manner that is not completely understood [28]–[31]. A better understanding of how thermal plasticity in body size, development time, and immature survival has evolved in *Drosophila* would shed light on the evolutionary ecology of the species. However, although the evolutionary history of the species is well studied at the phenotypic and genomic level, and interspecific observations of genome size-phenotype connections exist [10], genome size variation has not been considered in studies of the evolution of *D. melanogaster*.

Here, we take a quantitative genetic approach to address the issue of conspecific genome size variation and its life history consequences. We ask how an environmental variable, temperature, interacts with genome size to affect *D. melanogaster* development. Previous studies have measured intraspecific variation, focusing on geographic-dependent variation in a small set of intraspecific populations [1]–[5]. However, no studies to date have addressed the effects of intraspecific genome size variation on life history traits in a way that enables the measurement of phenotype in multiple environments for a large number of individual conspecific genotypes. Recently, such studies have been proposed to investigate the role of genome size on thermal plasticity [16].

The availability of sequenced inbred strains from Raleigh, NC natural populations (the *Drosophila melanogaster* Genetic Reference Panel, DGRP, https://www.hgsc.bcm.edu/content/drosophila-genetic-reference-panel [32], [33]) allows for replicated, accurate within-strain genome size estimates. In addition, the ease of measuring life history traits in these strains at different rearing temperatures makes *D. melanogaster* an ideal model organism for determining the relationships among genome size, temperature, and life history. Accordingly, we evaluated the extent of conspecific variation in genome size among 211 DGRP inbred strains, selected 50 lines representing the 25 of the largest and 25 smallest genomes, measured life history traits in all 50 of these lines, and asked if genome size variation correlates with variation in development-related life history traits or their environmental plasticity.

## Results

### Genome size varies significantly among inbred *Drosophila* lines

We quantified genome size in females for 211 DGRP lines using flow cytometry (Table 1, Table S1). We found considerable variation in average genome size among these strains, with the average genome size per strain ranging from a minimum of 169.7 Mbp and a maximum of 192.8 Mbp. The overall average genome size was 175.5 Mbp, which agrees well with the estimated genome size of 175 Mbp for the *y w* reference strain of *D. melanogaster* [34]. Further, the population appears to be biased toward the accumulation of large genomes (median = 175.1; skew = 0.5) [33]. Two of the strains, DGRP_378 and DGRP_554, were not included in the second release of the DGRP [33]. Interestingly, several of the large strains demonstrated instability in genome size, such that the addition of replicate measures did not reduce the within-strain standard error of genome size (Table 1).

**Table 1:** Variation in genome size in 50 selected DGRP strains with atypical genome sizes.

DGRP strain	*N*	Average genome size (Mb)	s.e.	Significance group(s)	Average genome size - mean	*t*-test (to mean) *P*-value
DGRP_38	6	192.8	10.1	A	17.3	0.15
DGRP_819	6	186.3	14.7	AB	10.8	0.52
DGRP_153	6	183.2	3.2	ABC	7.7	0.06
DGRP_40	6	181.2	1.1	BCD	5.7	0.004
DGRP_362	6	181.2	1	BCD	5.7	0.002
DGRP_892	6	181.1	3.3	BCD	5.6	0.15
DGRP_142	11	181.1	3.1	BCD	5.6	0.11
DGRP_517	11	181	0.7	BCD	5.5	0.00002
DGRP_837	5	181	0.7	BCD	5.5	0.001
DGRP_138	6	180.9	1.1	BCD	5.4	0.01
DGRP_93	6	180.8	1.1	BCD	5.3	0.01
DGRP_75	6	180.8	0.9	BCD	5.3	0.003
DGRP_26	6	180.8	1.4	BCD	5.3	0.02
DGRP_42	6	180.6	4.5	BCD	5.1	0.31
DGRP_391	6	180.4	1.3	BCD	4.9	0.01
DGRP_21	6	180.3	2.2	BCD	4.8	0.09
DGRP_45	6	180.2	0.7	BCD	4.7	0.001
DGRP_57	6	180.2	0.6	BCD	4.7	0.001
DGRP_69	6	180	0.4	BCD	4.5	0.0001
DGRP_101	6	179.9	0.8	BCD	4.4	0.002
DGRP_28	6	179.6	1.4	BCD	4.1	0.04
DGRP_705	6	179.5	1.2	BCD	4.0	0.02
DGRP_88	6	179.4	0.5	BCD	3.9	0.001
DGRP_790	6	179.2	1.1	BCD	3.7	0.03
DGRP_105	6	178.9	0.7	BCD	3.4	0.01
DGRP_820	3	172.9	0.7	CD	−2.6	0.06
DGRP_377	6	172.8	1.1	CD	−2.7	0.05
DGRP_307	3	172.7	0.3	CD	−2.8	0.01
DGRP_360	3	172.7	0.3	CD	−2.8	0.01
DGRP_441	6	172.7	0.7	CD	−2.8	0.01
DGRP_555	6	172.7	1	CD	−2.8	0.03
DGRP_786	5	172.6	0.6	CD	−2.9	0.01
DGRP_195	5	172.5	0.6	CD	−3	0.01
DGRP_237	7	172.5	0.4	CD	−3	0.0003
DGRP_379	3	172.5	0.2	CD	−3	0.004
DGRP_554	6	172.5	0.9	CD	−3	0.02
DGRP_787	6	172.5	0.7	CD	−3	0.01
DGRP_335	3	172.3	0.4	CD	−3.2	0.02
DGRP_406	5	172.3	0.7	CD	−3.2	0.01
DGRP_440	4	172.3	0.9	CD	−3.2	0.03
DGRP_313	3	172.2	0.7	CD	−3.3	0.04
DGRP_884	5	171.9	0.4	CD	−3.6	0.001
DGRP_321	5	171.7	0.9	CD	−3.8	0.01
DGRP_378	8	171.6	1	CD	−3.9	0.01
DGRP_332	5	171.2	0.8	D	−4.3	0.005
DGRP_595	5	171	0.5	D	−4.5	0.001
DGRP_318	3	170.5	0.1	D	−5	0.0003
DGRP_801	6	170.4	0.5	D	−5.1	0.0001
DGRP_181	5	170.1	0.9	D	−5.4	0.003
DGRP_208	10	169.7	0.3	D	−5.8	0.000000002

The mean genome size for *D. melanogaster* females is 175.5 Mb. *N* = Number of females. Mb = Megabase. s.e. = Standard error of the mean in Mb. Significance groups (A, B, C, D, etc.) differ at *P*<0.05 by a Duncan test. Average genome size - mean = deviation from the mean (175.5 Mb). *t*-test to the mean of 175.5 Mb determined significant (*P*<0.05) variation from the mean. Note that some strains were not significantly different from the mean due to their high variance in genome size.

To further demonstrate intraspecific variation in genome size, we performed a flow cytometry analysis with co-processed samples of a line with small genome size (DGRP_208, 169.7 Mb) and a line with large genome size (DGRP_517, 181 Mb) [35]. The histogram produced by co-processed females from these lines is shown in Figure S1. The co-preparations show separate fluorescence intensity peaks that differ in position precisely as expected from the genome size estimates (Table 1). Additional evidence of differences was provided by comparison of the proportion of under-replicated DNA in polytene tissues (Figure S2a,b) [36]. For the strains shown, DGRP_208 (169.7 Mb) and DGRP_517 (181 Mb), 88% of the DNA is fully replicated (12% unreplicated) in the smaller genome, while 86.2% of the DNA is fully replicated (13.8% unreplicated) in the larger genome. The 1.8% increase in the replicated sequences in the thorax represents 28% (3.18 Mb) of the 11.3 Mb difference between the strains; the remaining 72% (8.14 Mb) is under-represented in thoracic tissues suggesting a role for both fully replicated and under-replicated sequences in genome size expansion.

### *Drosophila* life history traits are associated with genome size

**Figure 1:**
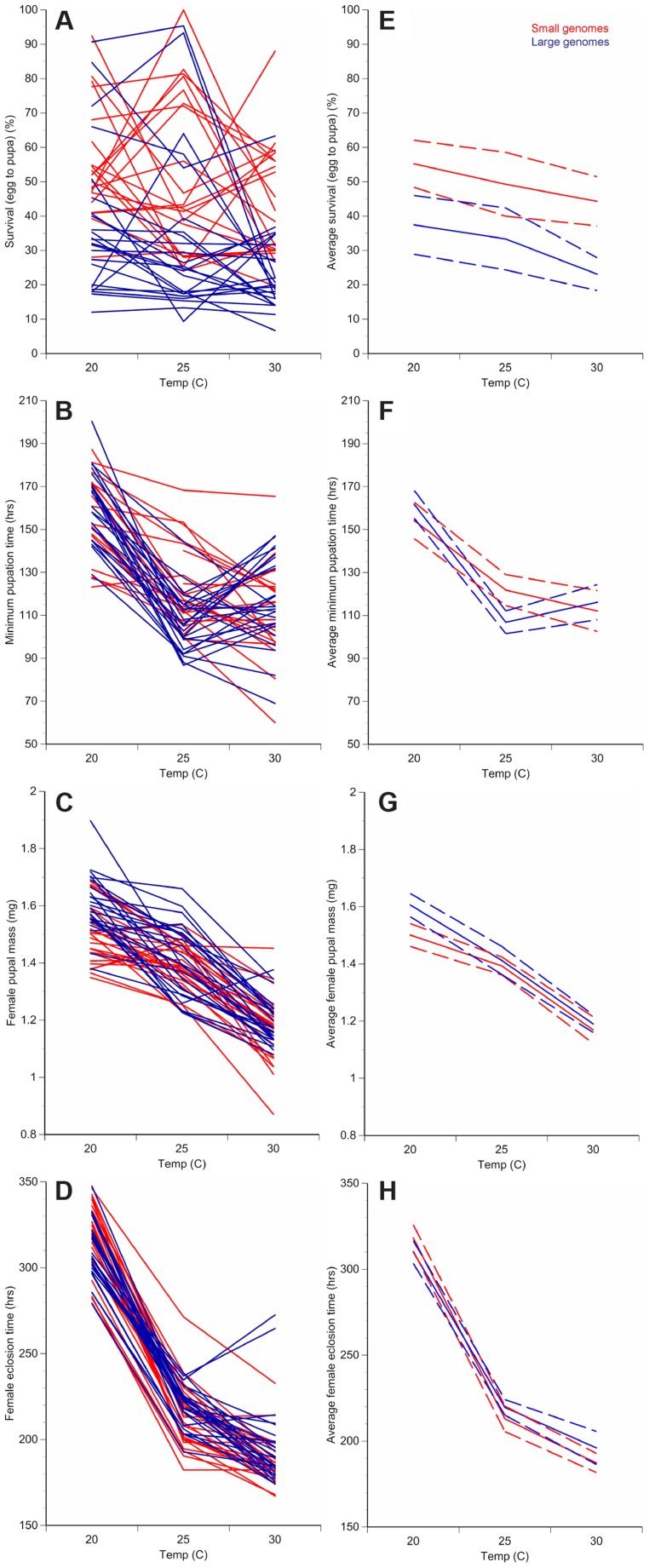
Changes in temperature alter development of *D. melanogaster* with small or large genomes. For each of the 25 small genome strains (red) and the 25 large genome strains (blue), the average survival to pupation (**A**), minimum pupation time (**B**), female pupal mass (**C**), and female eclosion time (**D**) was measured at 20°C, 25°C, and 30°C. Averaging the small genome strains (red) or the large genome strains (blue) for each phenotype shows temperature- and genome-size specific effects on survival to pupation (**E**), minimum pupation time (**F**), female pupal mass (**G**), and female eclosion (**H**). Increased genome size is associated with decreased survival to pupation (**A**, **E**) and increased female pupal mass (**C**, **G**). Dashed lines represent 95% confidence intervals.

**Figure 2:**
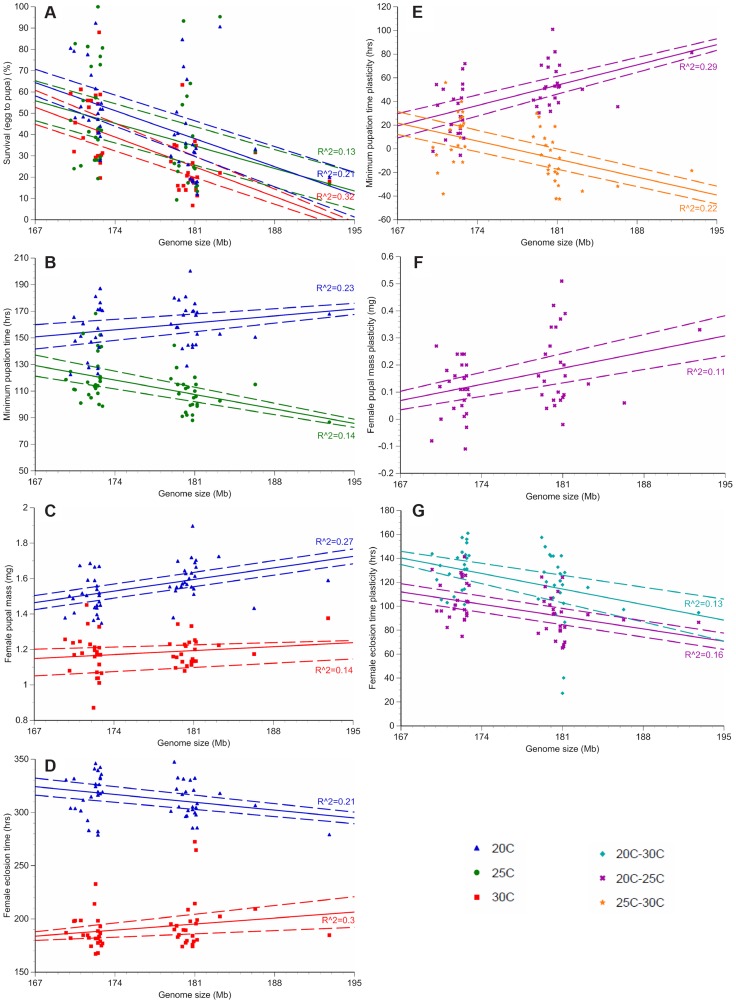
Genome size and temperature affect *D. melanogaster* development and plasticity. Only phenotypes with significant *R* ^2^ values (the proportion of variation in each phenotype due to genome size variation, not accounting for other genetic effects) are depicted. The average survival to pupation (**A**), minimum pupation time (**B**), female pupal mass (**C**) and female eclosion time (**D**) is shown for the 25 strains with the largest and smallest genomes at 20°C (blue triangle), 25°C (green circle), and 30°C (red square). Plasticity for minimum pupation time (**E**), female pupal mass (**F**) and female eclosion time (**G**) is shown between 20°C and 30°C (turquoise diamond), 20°C and 25°C (purple x) and 25°C and 30°C (orange star). Dashed lines represent 95% confidence intervals of the regression line.

In order to take advantage of the observed variation in genome size among inbred strains and examine the life history effects of an increase or decrease of genome size, we reared 25 strains with large female genomes and 25 strains with small female genomes (Table 1) at three temperatures (20°C, 25°C, and 30°C) and scored life history traits for each strain at each temperature (Figures 1, 2, S3; Table S1). A significance test across all genome size means of 211 strains derived from 1,052 measurements showed the 25 strains with the large genomes differed significantly from the 25 strains with the small genomes (Table 1) (*t*-test; *P*<0.001). The life history traits of survival to pupation, minimum pupation time, female pupal mass, and female eclosion time varied significantly across strains and temperatures (Figures 1, S3; Table S1). Survival to pupation strongly correlated with survival to adulthood (*r* = 0.975); therefore, we report only survival to pupation.

We fitted linear mixed models to the developmental phenotypes that included fixed effects of genome size, temperature, and the interaction between the two factors; as well as random effects accounting for additive and non-additive strain effects and the interaction between strain and temperature. We found substantial variation in the effect of genome size on the plasticity of all non-survival traits (Table 2). The magnitude and/or the direction of the effects of genome size on these phenotypes were dependent on the temperature, as evident by the significant interactions between genome size and temperature (Figure 2; Table 2). The effects of temperature on all four phenotypes were highly significant (*P* = 0.0013 for survival to pupation; *P*<0.0001 for minimal pupation time, female pupal mass, and female eclosion time). We further tested the effect of genome size on the four phenotypes for the three temperatures separately (Table 3). As expected when there is no interaction between genome size and temperature, the effect of genome size on survival to pupation was similar across all three temperatures (Table 3). On the other hand, genome size only affected the other traits in specific thermal conditions (Table 3). We also evaluated and visualized the significant relationships between phenotype and genome size on each temperature via simple regression of phenotypic line means on genome size (Figure 2, Table 4). The general conclusions from regressions did not vary if outlier lines with extremely large genomes (Table 1) were removed; therefore, all data were included in the analyses.

**Table 2:** Test for the effect of an interaction between genome size and temperature on life history traits.

Trait	Numerator df	Denominator df	F value	*P*-value
Survival from egg to pupa	2	560	0.20	0.8213
Minimum pupation time	2	566	6.98	0.0010
Female pupal mass	2	575	3.13	0.0446
Female eclosion time	2	675	6.03	0.0025

df: degrees of freedom.

**Table 3:** Effects of genome size on life history traits across three temperatures.

Trait	Temperature (°C)	Genome size effect (SE)	df	*t* value	*P*-value	*P*-value (with top 5 GWAS SNPs)	Variance explained by genome size
Survival from egg to pupa	20	−1.82 (0.544)	216	−3.35	0.001	0.0338	13.50%
	25	−1.52 (0.666)	131	−2.28	0.0244	0.1099	6.39%
	30	−1.97 (0.420)	214	−4.69	<0.0001	0.0002	23.03%
Minimum pupation time	20	0.70 (0.530)	216	1.31	0.1904	0.077	1.57%
	25	−1.58 (0.453)	135	−3.5	0.0006	0.053	17.20%
	30	0.46 (0.647)	216	0.72	0.4748	0.968	0.00%
Female pupal mass	20	0.0094 (0.00304)	192	3.1	0.0022	0.0128	13.78%
	25	0.0010 (0.00302)	192	0.34	0.7374	0.7292	0.00%
	30	0.0043 (0.00258)	192	1.66	0.0908	0.6829	3.19%
Female eclosion time	20	−1.10 (0.509)	271	−2.15	0.0322	0.4265	5.48%
	25	0.33 (0.444)	135	0.74	0.4577	0.1723	0.00%
	30	0.88 (0.579)	271	1.51	0.1312	0.4223	2.50%

**Table 4:** Results of regression analyses of thermal plasticity of life history traits by genome size.

Phenotype	20°C–30°C	20°C–25°C	25°C–30°C
Survival from egg to pupa	NS	NS	NS
Minimum Pupation Time	NS	***	***
Female Pupal Mass	NS	*	NS
Female Eclosion Time	**	**	NS

*P<0.05;

**P<0.01;

*** *P*<0.001;

NS = not significant *P*>0.05.

We estimated the proportion of phenotypic variation explained by genome size by comparing variance component estimates with or without genome size in the model for temperature/phenotype combinations where the effect of genome size was significant (Table 3). We found that genome size contributed between 6–23% of the total variation in survival to pupation (Table 3), 17% of the variation in minimum pupation time at 25°C, 14% of the variation in female pupal mass at 20°C, and 5% of the variation in female eclosion time at 20°C.

Given that genome size appeared to influence development in an environment-dependent manner, we derived a basic measure of the degree of plasticity in each phenotype and performed regressions of plasticity against genome size (Figure 2E–G, Table 4). There appears to be a complex relationship between genome size and plasticity, such that large genomes are more plastic or less plastic than small genomes, depending on the phenotype. For example, minimum pupation time showed genome size-dependent plasticity where large genomes were more responsive to 20°C to 25°C thermal shifts, whereas small genomes were more responsive to 25°C to 30°C shifts. For the most genome size-sensitive phenotypes (e.g. survival to pupation) thermal plasticity was relatively independent of genome size.

We further assessed correlations among all the phenotypes and genome size using a principal component (PC) analysis (Table S2). The first two PCs partitioned the data on the basis of genome size and accounted for 21% and 16%, respectively, of the total variation observed. The loadings of the first two PCs reflected the correlation of genome size with phenotype (genome size correlation to PC1 and PC2 was −0.23 and 0.18, respectively), and they correctly partitioned all but a few lines into large or small genome size groupings. Thus PC analyses upheld the general inferences obtained from the mixed model analysis.

**Figure 3:**
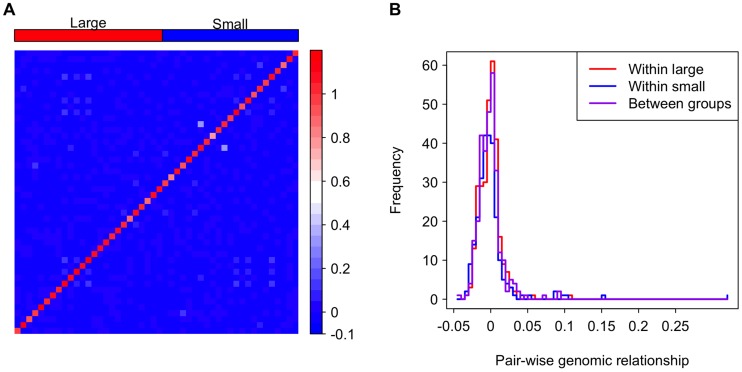
Genomic relationship among and between the large and small genome groups. (**A**) A heat map depicting the genomic relationship between DGRP lines. Genome size is ordered decreasingly from left to right and bottom to top. The strains in the groups are indicated as the red and blue rectangles on the top of the heat map. (**B**) Histograms showing genomic relationships within and between the genome size groups.

It is possible that genotype is confounded with genome size. For example, if co-adapted suites of traits are associated with specific chromosomes of different sizes, strains with small genome sizes may also have distinct genotypically correlated phenotypes. If this is the case, we expect lines within the large or small genome groups would be more closely related to each other than lines between the groups. Indeed, genome size is significantly correlated with inversion karyotypes in the DGRP, and lines with the same inversion karyotypes are slightly more related to each other [33]. However, inversions clearly do not completely explain genome size variation, accounting for only ∼0.5 Mb of the variation in genome size [33]. To address the concern of relatedness among strains of atypical genome size, we evaluated the pair-wise genomic relatedness among lines. Relatedness between lines within the large and small genome size groups is not higher than that between groups, suggesting that the large and small genome lines form a genetically homogeneous pool rather than two separate clusters (Figure 3). This analysis, in combination with the fact that the aforementioned mixed models were designed to account for any confounding cryptic relationship among the lines, clearly suggests that there are correlations of genome size with life history traits that are independent of potential confounding genotypic effects at a broad genome-wide scale.

Finally, to assess whether pleiotropic effects of QTLs affecting both genome size and the phenotypes could explain the observed correlation between genome size and life history traits, we tested the effect of genome size conditional on the top five genetic variants associated with genome size detected by a genome wide association study (GWAS) [33]. Although the inclusion of top GWAS hits diminished the significance of the association between genome size and life history traits (Table 3), this result is expected when there is a genuine association between genome size and life history traits. Inclusion of the top GWAS hits does not fully explain the effects of genome size on life history traits and actually lowered the *P*-values for genome size associations at some temperatures.

### Genome size and gene expression

**Figure 4:**
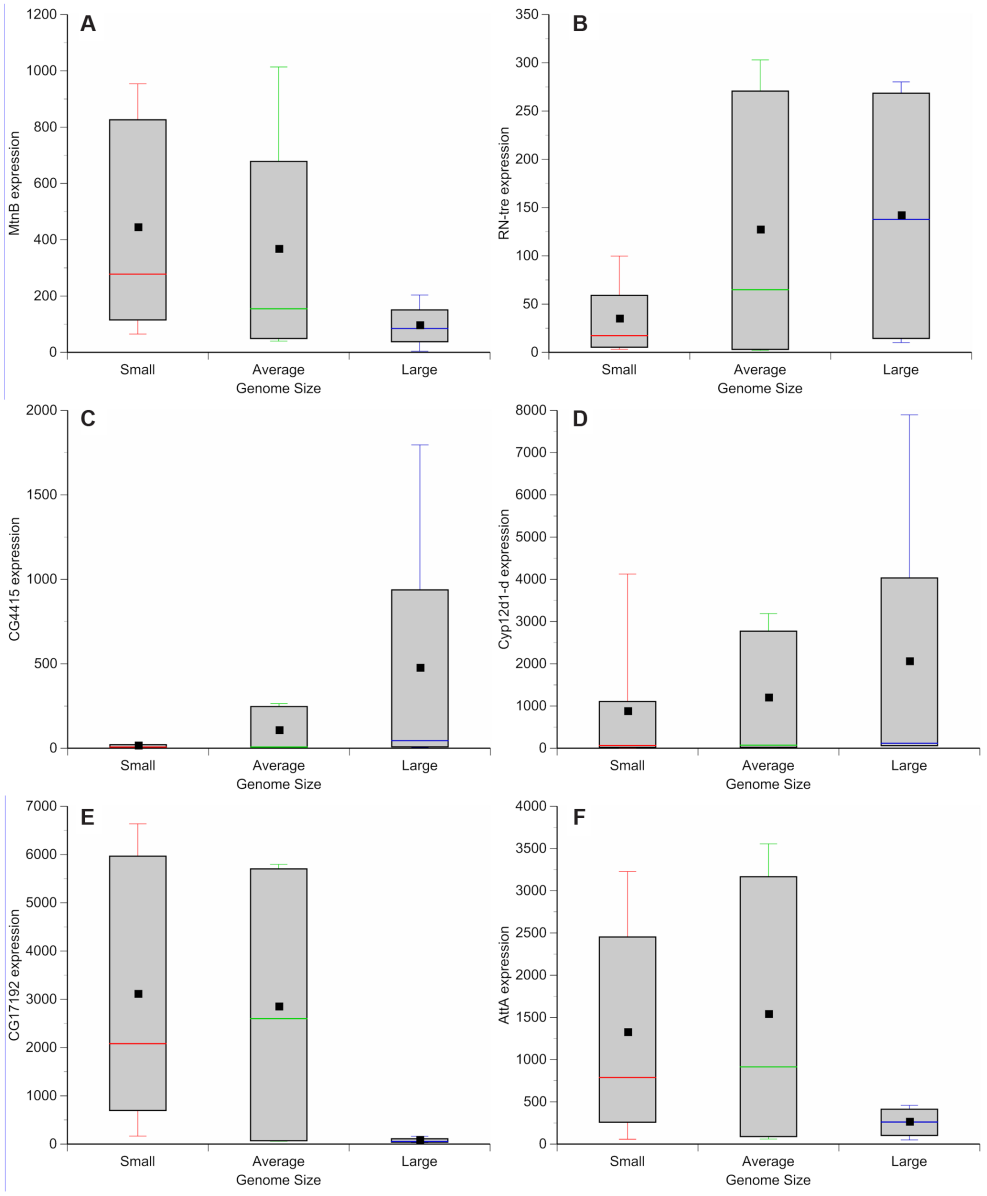
Genome size-associated changes in gene expression. Box plots highlight the most up-regulated and down-regulated genes based on comparisons between small (red), average (green), and large (blue) genome strains. (**A**) *MetallothioneB* (*MtnB*) (**B**) *Related to the N terminus of tre oncogene* (*RN-tre*). (**C**) *CG4415*. (**D**) *Cyp12d1-d*. (**E**) *CG17192*. (**F**) *AttacinA* (*AttA*).

Genome wide variation in gene expression has been evaluated using microarrays for a subset of the DGRP strains [37]. We assessed whether there is variation in gene expression between lines with small and large genomes. These observations can be used to guide further efforts to dissect mechanisms by which genome size can lead to phenotypic differences. Comparisons between microarray results of adult females of small genome (DGRP_208, DGRP_307, DGRP_313, DGRP_335, DGRP_360, DGRP_379, DGRP_555, DGRP_786, and DGRP_820), large genome (DGRP_362, DGRP_391, DGRP_517, DGRP_705), and more species-typical genome (DGRP_301, DGRP_303, DGRP_304, DGRP_306, DGRP_315, DGRP_324, DGRP_357, DGRP_358, DGRP_365, DGRP_375, DGRP_380, DGRP_399, DGRP_427, DGRP_437, DGRP_486, DGRP_514, DGRP_639, DGRP_707, DGRP_712, DGRP_714, DGRP_730, DGRP_732, DGRP_765, DGRP_774, DGRP_799, DGRP_852, DGRP_859) strains revealed 562 differentially expressed genes (Figure 4, Tables S3, S4, S5, S6, S7, S8, S9, S10, S11, S12, S13). One hundred forty-nine genes were up-regulated in strains with small genomes (Figure 4; Table S3); 227 genes were up-regulated in strains with large genomes (Figure 4; Table S4). Strains with small genomes down-regulated 91 genes (Figure 4, Table S5) while strains with large genomes down-regulated 95 genes (Figure 4, Table S6). Gene ontology enrichment analyses revealed that strains with small genomes up-regulated genes related to metabolism, mitosis, egg development, translation, and salt transport (Tables S7, S9) and down-regulated genes related to development and enzymatic activity (Table S11). The up-regulated genes included ion binding genes that appear to be differentially regulated during exposure to thermal and chemical environments that affect oxidative stress [38]. Strains with large genomes up-regulated genes involved with development, metabolism, TOR signaling, and heme and ion binding (Tables S8, S10) while down-regulating primarily genes affecting gametogenesis (Tables S12, S13). Many of the enriched genes were expressed in the digestive system. This suggests, (in combination with the increased expression of metabolism and TOR signaling genes in strains with large genomes), that nutritional ecology plays an important role in these responses.

*Drosophila* chromosomal inversions are classically understood to clinally segregate in an adaptive fashion [39], [40] and some of the phenotypes we studied can vary along clines [23]–[27]. Of the strains evaluated for gene expression, none of the large genomes contained known inversions (Table 5). In the expression comparison, inversions do not obviously explain differences in gene expression between large and small genomes.

**Table 5:** Chromosomal inversions in the 50 lines evaluated for thermal responses of life history and gene expression.

DGRP strain	Chromosome *2* Inversions	Chromosome *3* Inversions	Average genome size (Mb)
DGRP_38		*In(3R)K* [het]	192.8
DGRP_837	*In(2L)t*		181.0
DGRP_138		*In(3R)P*	180.9
DGRP_93	*In(2L)t*		180.8
DGRP_26	*In(2L)t*		180.8
DGRP_69	*In(2R)NS*	*In(3L)Y* [het]	180.0
DGRP_101	*In(2L)t* [het]		179.9
DGRP_28	*In(2R)NS*		179.6
DGRP_88	*In(2L)t* [het]		179.4
DGRP_105		*In(3R)K*	178.9
DGRP_820*		*In(3R)Mo*	172.9
DGRP_377	*In(2L)t* [het]; *In(2R)Y2* [het]; *In(2R)NS* [het]		172.8
DGRP_555*		*In(3R)Mo*	172.7
DGRP_786*		*In(3R)P*	172.6
DGRP_554		*In(3R)Mo*	172.5
DGRP_237	*In(2L)t* [het]; *In(2R)NS* [het]		172.5
DGRP_406			172.3
DGRP_335*		*In(3R)Mo* [het]	172.3
DGRP_440		*In(3R)K* [het]	172.3
DGRP_313*	*In(2L)t*		172.2
DGRP_884		*In(3R)P* [het]	171.9
DGRP_595	*In(2L)t* [het]		171.0

Data are from Ref [33]. [het] = heterozygous for the inversion; K = Kodani; Mo = Missouri; NS = Nova Scotia; P = Payne; Y = Yutaka; Y2 = Yutaka #2.

* indicates strains that were used in gene expression analyses.

## Discussion

The role of natural selection in the evolution of genome size evolution is hotly debated. If natural selection directly or indirectly affects the evolution of genome size, genomes must vary conspecifically and be connected to adaptive phenotypes like life history traits. By measuring genome sizes of 211 inbred *D. melanogaster* strains derived from a single population, we document the presence of conspecific genome size variation, and the association of genome size with several life history traits in strains with the most extreme genome sizes. Only a few previous studies examined the correlation between genome size and organismal phenotypes [1], [5]. Here, we provide evidence of complex correlations between genome size and multiple life history traits in an experiment that affords greater resolution of genome size – phenotype connections than is possible with studies of wild individuals (Figures 1, 2).

A major conclusion of our study is that genome size appears to contribute a significant proportion of variation in life history traits in an environmentally dependent manner (Figures 1–2, Tables 2–4, S2). Genome size effects ranged from 5%–23% depending on the trait and temperature. These estimates were obtained after accounting for the additive and non-additive genotypic effects of strains. PC analyses uphold the general interpretations of the linear models and separate the phenotypic data based on genome size, with the first two principal components correlating with genome size. This study was not designed to infer the mechanisms or nature of the plastic responses, only to demonstrate their existence. More detailed studies investigating the details of this phenomenon are warranted.

Future efforts should be targeted toward understanding the degree to which genome size effects are rooted in the “quality” versus “quantity” of the genome. While the reported results could be due to molecular changes in metabolism necessary to maintain a larger genome, these metabolic effects cannot currently be definitively disentangled from the fact that they could be associated with adaptive chromosomes of different sizes (such as the inversions on chromosome *3R* in the small genomes). However, the fact that our analyses accounted for genomic levels of relatedness among the studied strains suggest that both genome size and genotypes of strains with the largest and smallest genome sizes contribute to variation in our target phenotypes.

We assessed the effect of female genome size on female-specific (pupal mass and eclosion time) and non-sex-specific (pupal survival, minimum pupation time, and adult survival) traits. Thus, it is possible (depending on the mechanism of our observations) that females and males have divergent genome size-dependent phenotypic responses. It should be noted that, since females and males exhibit sexually dimorphic life history traits [41]–[44], which can have a different optimum for each sex, it will be interesting to assess whether dimorphism in genome size exists and if it is a mechanism by which the sexes can manage conflicts in life history trait optima.

The DGRP consortium conducted a genome wide association study on genome size with the data produced by this project, along with a suite of complex quantitative traits [33]. Using linear mixed models, relatedness among individuals in genome-wide association studies is accounted for by estimating average levels of genomic similarity, before genetic associations with phenotype are identified [45]. Interestingly, variation in genome size is correlated with inversions in the DGRP, and correction for inversion karyotype associations resulted in the identification of several strong associations of genetic variants with genome size. When there is evidence of a genome size effect on a phenotype, the results of our work suggest that it may be appropriate to incorporate genome size into mapping efforts. Potential correction for genome size effects in mapping experiments may include using genome size as a cofactor (as observed in [45] for alleles of the *frigida* locus in *Arabidopsis thaliana*) and incorporating genome size as a correlated phenotype (as described in [46]).

In theory, inbreeding should have just partitioned genome size variation among the strains of the DGRP, revealing genome size variation in a manner that allowed us to repeatedly sample genome size and phenotype from the same genotype. However, one could imagine that inbreeding might itself be a cause of genome size variation, which is a caveat that must be considered in this experiment. As a consideration to the strains analyzed, given the bias in strain maintenance (healthy strains were maintained preferentially), an inbreeding effect on genome size should be limited in its effect on fitness. In addition, if genome size shifted with the creation of the strains, it must be remembered that there were deviations from the average genome size in both directions. This would indicate support for Lynch's proposal that genome size evolution is due to genetic drift [15], [19]. However, given our observation that variation in genome size is associated with several life history traits, we speculate that variation in genome size created by neutral processes may be reinforced in some instances by non-neutral forces.

It is also possible that inbreeding could result in the fixation of alleles that pleiotropically affect life history traits and genome size. Such pleiotropic effects could drive an association between genome size and life history traits. Indeed, top GWAS hits of genome size variation explained some, but not all, of the association between genome size and life history traits (Table 3). However, this is a necessary statistical outcome even when the variation in the life history traits is entirely caused by genome size variation. In fact, in the event that genome size is causal for the life history traits, any QTL for genome size would appear to be pleiotropic for the life history traits. Whether the variation in life history traits is caused independently by the pleiotropic QTLs or by variation in genome size must be addressed by breaking the pleiotropic QTLs into independent ones, which may or may not be possible and is beyond the scope of the current study.

In conclusion, we observed significant variation in genome sizes among sequenced *D. melanogaster* strains; and large and small genome sizes correlated with conspecific variation in life history traits. These results indicate that a portion of phenotypic variation may be due to genome size effects (potentially up to 23%, in a trait and environment dependent manner). What is even more interesting is that genome size variation appears to be associated with phenotypic plasticity in several traits, suggesting that the evolution of genome size may produce novel correlations among life history traits in a temperature-dependent manner. These observations support the recently proposed link between genome size and thermal plasticity [16] and advance our understanding of life history trait correlations. This research indicates that studies of genome size evolution can contribute to two major problems in biology: elucidating the genetic architecture of complex phenotypes and identifying mechanisms of life history trait evolution.

## Materials and Methods

We examined 211 *D. melanogaster* strains obtained from the *Drosophila melanogaster* Genetic Reference Panel (https://www.hgsc.bcm.edu/content/drosophila-genetic-reference-panel) and Bloomington *Drosophila* Stock Center (flystocks.bio.indiana.edu). Of these 205 are reported in the most recently released genomic data for the DGRP [33]. Stocks were maintained at room temperature on Bloomington's standard medium (The Bloomington *Drosophila* Stock Center, Indiana University; Table S14).

### Measuring, testing, and verification of genome size differences

We estimated genome size of 1,052 individual females from the 211 inbred *D. melanogaster* strains using flow cytometry, using *D. virilis* (1C = 328 Mb) as an internal standard. The final concentration of propidium iodide stain was 25 µg/mL [47]. In brief, samples were prepared from a single adult female head that was homogenized in Galbraith buffer using a Dounce tissue grinder and nylon mesh filtration. Samples were incubated at 4°C for approximately 30–60 minutes in 25 µg/mL propidium iodide. Flow cytometry measured 1,000 cell counts per unknown and control sample. Genome size of the unknown = GS_control_×PI−fluor_unknown_/PI-fluor_control_ where PI-fluor is the channel number of red propidium iodide (PI) fluorescence [47]. Mean genome size averages were compared using Proc GLM with Duncan multiple range tests in SPSS (SPSS Inc. Version 16.0, Chicago, IL) and *t*-test comparisons to the population mean. Genome size differences between large and small genome strains were verified [20] by co-preparation of an individual from a high (DGRP_517) with one from a low (DGRP_208) genome size line. The extent of under-replication in polytene tissues of high and low genome size lines was scored using thoracic tissues prepared as described for genome size estimates [36].

### Assessing developmental phenotypes

In order to maximize the variation in genome size and the phenotypic variation, 25 strains with large female genomes (DGRP_21, DGRP_26, DGRP_28, DGRP_38, DGRP_40, DGRP_42, DGRP_45, DGRP_57, DGRP_69, DGRP_75, DGRP_88, DGRP_93, DGRP_101, DGRP_105, DGRP_138, DGRP_142, DGRP_153, DGRP_362, DGRP_391, DGRP_517, DGRP_705, DGRP_790, DGRP_819, DGRP_837, and DGRP_892) and 25 strains with small female genomes (DGRP_181, DGRP_195, DGRP_208, DGRP_237, DGRP_307, DGRP_313, DGRP_318, DGRP_321, DGRP_332, DGRP_335, DGRP_360, DGRP_377, DGRP_378, DGRP_379, DGRP_406, DGRP_440, DGRP_441, DGRP_554, DGRP_555, DGRP_595, DGRP_786, DGRP_787, DGRP_801, DGRP_820, and DGRP_884) [33] were chosen for phenotypic analysis. Male and female flies from these strains were passaged to perforated egg-laying bottles with a 35 mm grapefruit plate (10% grapefruit juice, 1% EtOH) and provided a small amount of yeast paste. Oviposition occurred at room temperature. Eggs were collected two hours after introduction of females. Seventy-five eggs were placed in vials containing Bloomington's standard medium (The Bloomington *Drosophila* Stock Center, Indiana University; Table S14) for all experiments. Vials were placed in a 20°C, 25°C, or 30°C incubator under a 12-hr light∶dark cycle with 70% humidity. Ten replicate vials were set up for each strain at each temperature; three vials were used to measure pupal phenotypes (survival from egg to pupa, minimum pupation time, and female pupal mass) and three vials were used to measure adult phenotypes (survival from egg to adult, and female eclosion time). Survival to pupation or adulthood was calculated as the number of total pupae or adults produced, respectively, divided by 75, the number of eggs in each vial. Vials with high egg mortality, which was rare, were not used in calculating survivorship. Minimum pupation time was measured as the time elapsed from when eggs were placed into the vial until the emergence of the first pupal case at an 8 hour temporal resolution. A total of 50 females (10 per vial) per strain at each temperature were weighed individually to calculate female pupal mass. Eclosion was recorded at 8:00 AM, 2:00 PM, and 8:00 PM each day to calculate the average eclosion time of each female in each vial.

For each phenotype, the significance of genome size and temperature effects, as well as their interaction, was determined using the MIXED procedure in SAS. We first assessed the significance of genome size by temperature interaction by fitting the following model, *y* = μ+*g*+*T*+*g:T*+*s*+*S*+*S:T*+*e*, where *y* is the phenotype being modeled, μ is the overall mean, *g* is the fixed effect of genome size of the strain, *T* is the fixed effect of temperature on which the flies are raised, *g:T* is the interaction between genome size and temperature, *s* is the random additive genetic effect with the covariance matrix determined by the pair-wise genomic relationships between strains, *S* is the random strain effect which accounts for additional variation between strains, *S:T* is the interaction between strain and temperature, and *e* is the residual. When testing the effect of genome size on life history traits in the three temperatures separately, a reduced model was fitted, *y* = μ+*g*+*s*+*S*+*e* and the effect of genome size was tested by type III F test. We also tested the effect of genome size on life history traits conditional on the five most significant genetic variants associated with genome size variation in a GWAS (X_21136189_SNP, 3L_5383897_SNP, 2L_6541787_SNP, 2L_6035179_SNP, 3R_19140723_SNP) [33] by including their genotypes in the model as fix effects.

Plasticity was scored three ways. First, we subtracted the mean of each phenotype from each strain at 30°C from the phenotype of that strain at 20°C. Second, we subtracted the mean of each phenotype from each strain at 25°C from the phenotype of that strain at 20°C. Finally, we subtracted the mean of each phenotype from each strain at 30°C from the phenotype of that strain at 25°C. This resulted in 50 measurements for each metric of pair-wise plasticity, which were regressed against genome size using Linear Regression in SPSS (SPSS Inc. Version 16.0, Chicago, IL). All of the aforementioned measures were done with line means as genome size and phenotype were not scored in the same individuals. Finally, PC analyses were also performed using SAS software to assess correlations among the phenotypes and genome size.

### Evaluating relatedness within and between strains with large and small genomes

Pairwise relatedness was extracted from the second release of genomic data from the DGRP [33]. Genome-wide levels of relatedness were calculated for all of the strains in that project, including some that have not been identified for further analyses because they exhibited signatures of relatedness to others whose genomes had already been sequenced [48]. Reported here are the relevant levels of genomic relatedness among the strains of atypical genome size. These values were used to generate Figure 3.

### Assessing genome size-specific gene expression

A previous study used microarray analysis to determine gene expression changes in a subset of the DGRP lines [37]. Briefly, RNA was extracted from two independent pools of 25 three to five day old flies per sex per line during the same two hour window each day. They were only evaluated in one environment. RNA extraction, labeling, and hybridization was randomized, and normalized values of gene expression were determined using median standardization [37]. We focused on the female microarrays. Of the 40 strains analyzed, nine had small genomes (DGRP_208, DGRP_307, DGRP_313, DGRP_335, DGRP_360, DGRP_379, DGRP_555, DGRP_786, DGRP_820), four had large genomes (DGRP_362, DGRP_391, DGRP_517, DGRP_705), and the remaining 27 had average-sized genomes (DGRP_301, DGRP_303, DGRP_304, DGRP_306, DGRP_315, DGRP_324, DGRP_357, DGRP_358, DGRP_365, DGRP_375, DGRP_380, DGRP_399, DGRP_427, DGRP_437, DGRP_486, DGRP_514, DGRP_639, DGRP_707, DGRP_712, DGRP_714, DGRP_730, DGRP_732, DGRP_765, DGRP_774, DGRP_799, DGRP_852, DGRP_859).We extracted expression values for each strain (averaged across each strain's replicates) using the PM-MM algorithm of dChip (one nucleotide between the probe and target sequence is mismatched) [49]. We focused on genes with expression levels greater than 50. We used cyber-T Bayesian *t*-tests [50] (*P*<0.05) and false discovery rate [51] (FDR<0.05) analyses to determine significant changes in gene expression. Genes that were identified as being up-regulated in small genomes showed increased expression in strains with small genomes compared to 1) strains with average genomes and 2) strains with large genomes. Genes were deemed as down-regulated in small genomes when they were down-regulated in strains with small genomes compared to 1) strains with average genomes and 2) strains with large genomes. We followed similar criteria for up or down-regulated genes in large genomes. We assessed significant enrichment of gene ontology terms using DAVID Functional Annotation Tool [52], [53] and GO Finder [54]. Each gene list (up-regulated in small genomes, down-regulated in small genomes, up-regulated in large genomes, and down-regulated in large genomes) was compared independently to the *D. melanogaster* genome to assess enrichment (*P*<0.05) of biological processes, cellular components, and molecular functions.

## Supporting Information

Figure S1 Large and small genomes have distinct genome size differences. Co-preparations of individuals from a strain with large genome size and a strain with small genome size show two distinct genome size peaks. Each peak is in position expected for the respective strains, confirming the differences between genome sizes.
(TIF)

Figure S2 Euchromatin levels vary between small and large genomes. Total DNA differences between strains include sequences that are and are not replicated in polytene thoracic tissues. Eighty-eight percent of the DNA is replicated in the smaller genome (DGRP_208) (**A**), while 86.2% of the DNA is replicated in the larger genome (DGRP_517) (**B**).
(TIF)

Figure S3 Box plots depicting genome size and temperature effects on *D. melanogaster* development. Survival (egg to pupa) (**A**), minimum pupation time (**B**), female pupal mass (**C**), and female eclosion time (**D**) is shown for the small and large genome size strains at 20°C (green), 25°C (blue) and 30°C (red).
(TIF)

Table S1 Genome size and phenotype data for the experiment.
(XLSX)

Table S2 Results from a principal component analysis of the phenotype and genome size data.
(DOCX)

Table S3 Genes up-regulated in strains with small genomes.
(XLSX)

Table S4 Genes up-regulated in strains with large genomes.
(XLSX)

Table S5 Genes down-regulated in strains with small genomes.
(XLSX)

Table S6 Genes down-regulated in strains with large genomes.
(XLSX)

Table S7 Gene ontology analysis results from DAVID of genes up-regulated in small genomes.
(XLSX)

Table S8 Gene ontology analysis results from DAVID of genes up-regulated in large genomes.
(XLSX)

Table S9 Gene ontology analysis results from GOFinder of genes up-regulated in small genomes.
(XLSX)

Table S10 Gene ontology analysis results from GOFinder of genes up-regulated in large genomes.
(XLSX)

Table S11 Gene ontology analysis results from DAVID of genes down-regulated in small genomes.
(XLSX)

Table S12 Gene ontology analysis results from DAVID of genes down-regulated in large genomes.
(XLSX)

Table S13 Gene ontology analysis results from GOFinder of genes down-regulated in large genomes.
(XLSX)

Table S14 *Drosophila* diet composition.
(DOCX)
