# Supplementary figures and images for: Intrapopulation Genome Size Variation in D. melanogaster Reflects Life History Variation and Plasticity
Source: PLoS Genet. 2014 Jul 24;10(7):e1004522. doi: 10.1371/journal.pgen.1004522 (PMC4109859; doi:10.1371/journal.pgen.1004522)

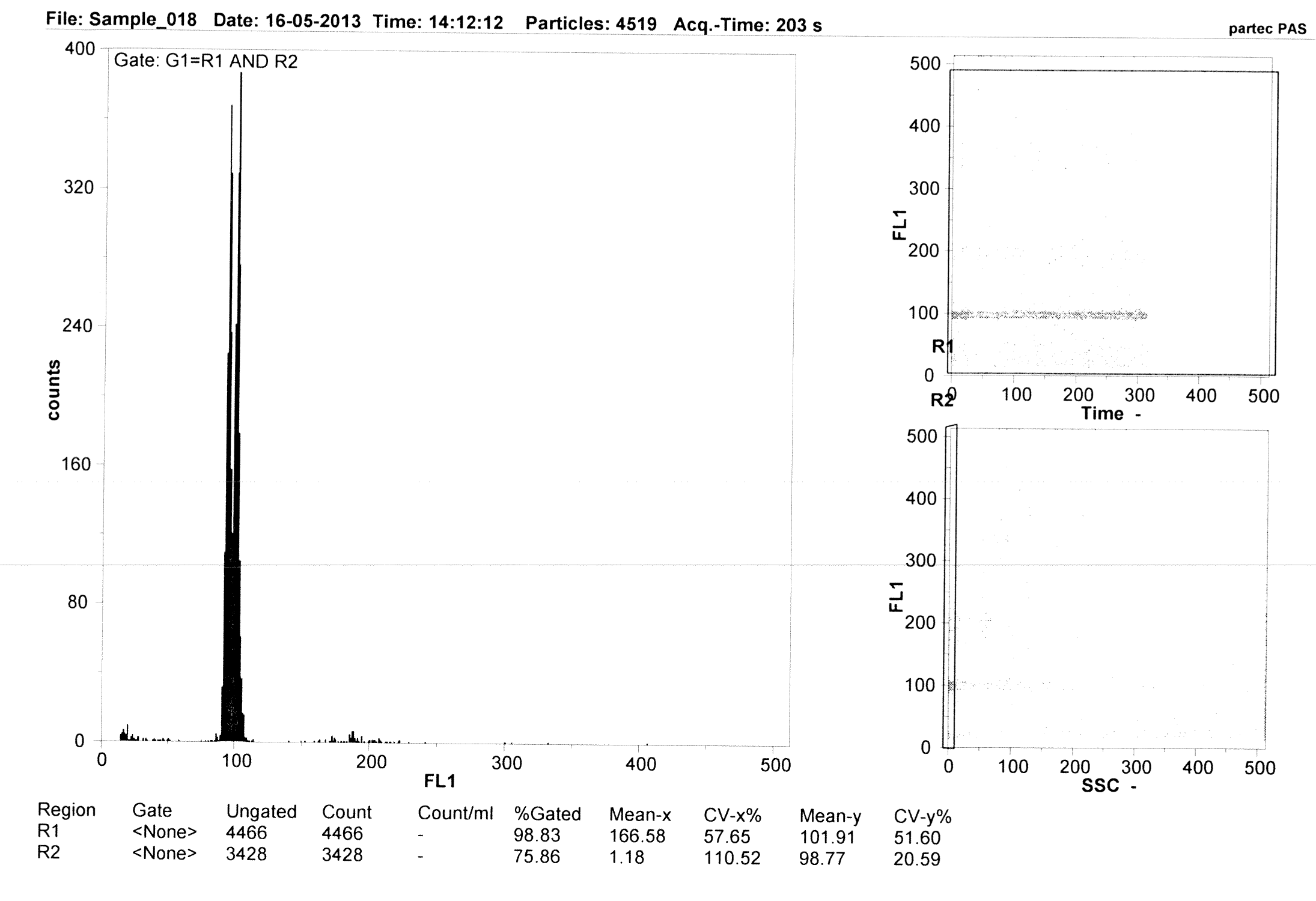

Supplement: Figure S1 — Large and small genomes have distinct genome size differences. Co-preparations of individuals from a strain with large genome size and a strain with small genome size show two distinct genome size peaks. Each peak is in position expected for the respective strains, confirming the differences between genome sizes. (TIF) [file pgen.1004522.s001.tif]

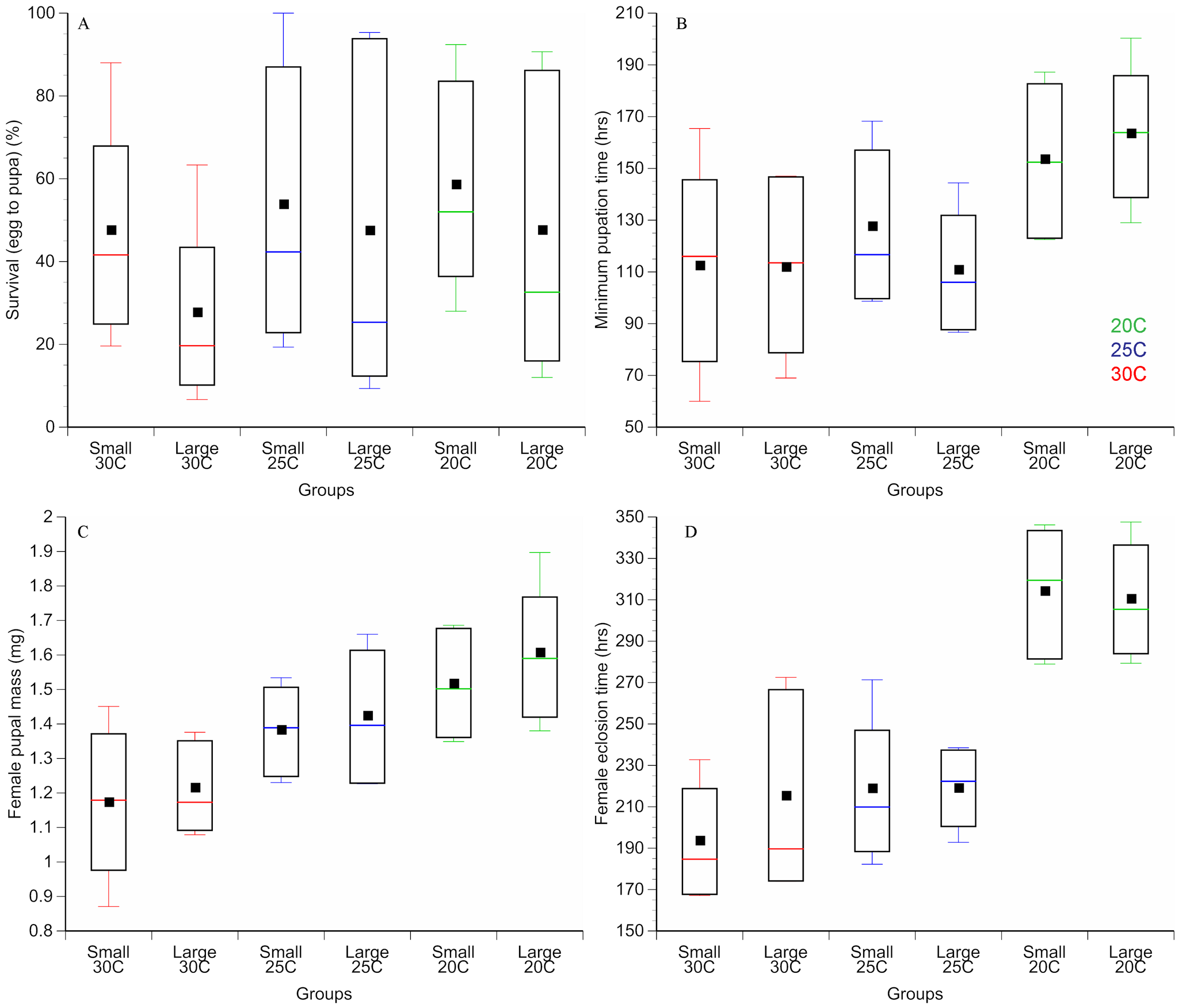

Supplement: Figure S3 — Box plots depicting genome size and temperature effects on D. melanogaster development. Survival (egg to pupa) (A), minimum pupation time (B), female pupal mass (C), and female eclosion time (D) is shown for the small and large genome size strains at 20°C (green), 25°C (blue) and 30°C (red). (TIF) [file pgen.1004522.s003.tif]
