# Supplementary material for: Intrapopulation Genome Size Variation in D. melanogaster Reflects Life History Variation and Plasticity
Source: PLoS Genet. 2014 Jul 24;10(7):e1004522. doi: 10.1371/journal.pgen.1004522 (PMC4109859; doi:10.1371/journal.pgen.1004522)
Supplement: Table S2 — Results from a principal component analysis of the phenotype and genome size data. (DOCX) [file pgen.1004522.s005.docx]

**Table S2.**

Genome Size Data 11:10 Thursday, October 9, 2008 649

The PRINCOMP Procedure

Observations 45

Variables 31

Simple Statistics

Mb SA30 SA25 SA20 SP30 SP25

Mean 176.9222222 26.11400000 39.75422222 43.64555556 32.97777778 41.70622222

StD 5.1290567 14.68430677 25.39968472 20.87539641 17.76680036 24.87980646

Simple Statistics

SP20 MINP30 MINP25 MINP20 FPM30 FPM25

Mean 45.72444444 114.2480000 114.4382222 158.1911111 1.187955556 1.405133333

StD 20.87507872 21.1699060 18.6071066 18.1521741 0.091324585 0.105496575

Simple Statistics

FPM20 FET30 FET25 FET20 SAP2025 SAP2530

Mean 1.566955556 190.7455556 217.6500000 311.9406667 3.89133333 13.64022222

StD 0.110760790 20.8125391 16.0802256 18.0635316 23.09843487 27.26018137

Simple Statistics

SAP2030 SPUPP2025 SPUPP2530 SPUPP2030 MINPTP2025 MINPT2530

Mean 17.53155556 4.01822222 8.72844444 12.74666667 43.75311111 0.19022222

StD 17.73827051 22.77949929 25.96274770 19.95035315 23.50959416 23.72767022

Simple Statistics

MINPT2030 FPMP2025 FPMP2530 FPMP2030 FETP2025 FEP2530

Mean 43.94311111 0.1613333333 0.2175555556 0.3791111111 94.29177778 26.90471051

StD 25.87354834 0.1302724418 0.1051223433 0.1357441146 16.05943992 19.04162254

Simple Statistics

FEP2030

Mean 121.1955556

StD 27.2895167

Genome Size Data 11:10 Thursday, October 9, 2008 650

The PRINCOMP Procedure

Correlation Matrix

Mb SA30 SA25 SA20 SP30 SP25 SP20 MINP30 MINP25

Mb 1.0000 -.4764 -.3376 -.4253 -.5773 -.3436 -.4620 0.1141 -.4693

SA30 -.4764 1.0000 0.1577 0.5493 0.7510 0.1700 0.5190 -.2077 0.1238

SA25 -.3376 0.1577 1.0000 0.5162 0.2919 0.9977 0.5123 -.2455 -.0615

SA20 -.4253 0.5493 0.5162 1.0000 0.4152 0.5208 0.9724 -.0933 0.1174

SP30 -.5773 0.7510 0.2919 0.4152 1.0000 0.2948 0.4764 -.3632 0.1030

SP25 -.3436 0.1700 0.9977 0.5208 0.2948 1.0000 0.5159 -.2533 -.0555

SP20 -.4620 0.5190 0.5123 0.9724 0.4764 0.5159 1.0000 -.0938 0.1732

MINP30 0.1141 -.2077 -.2455 -.0933 -.3632 -.2533 -.0938 1.0000 0.2937

MINP25 -.4693 0.1238 -.0615 0.1174 0.1030 -.0555 0.1732 0.2937 1.0000

MINP20 0.2116 -.5575 -.1819 -.3163 -.4578 -.1903 -.3367 0.1408 0.1821

FPM30 0.1413 -.0275 -.1214 -.0620 -.1441 -.1148 -.0630 -.0045 -.1840

FPM25 0.0048 -.1727 -.0232 -.0854 -.0006 -.0291 -.0988 -.0020 -.1943

FPM20 0.3521 -.1621 -.1664 0.0007 -.3148 -.1660 -.0943 0.2683 -.3613

FET30 0.2131 -.1630 -.1970 -.2036 -.0727 -.2189 -.1478 0.4549 0.1908

FET25 0.0803 -.3197 -.2786 -.1127 -.3132 -.2810 -.1337 0.5745 0.4661

FET20 -.2457 -.1183 0.0797 0.2969 -.0012 0.0768 0.2897 0.0670 0.4409

SAP2025 -.0132 0.3230 -.6331 0.3362 0.0543 -.6265 0.3154 0.1857 0.1737

SAP2530 -.0579 -.3917 0.8468 0.1851 -.1326 0.8381 0.1978 -.1169 -.1240

SAP2030 -.1062 -.1814 0.4769 0.7221 -.1331 0.4722 0.7147 0.0621 0.0356

SPUPP2025 -.0481 0.2900 -.6202 0.3223 0.1147 -.6194 0.3529 0.1907 0.2194

SPUPP2530 0.0658 -.3511 0.7564 0.2149 -.4018 0.7566 0.1683 0.0058 -.1237

SPUPP2030 0.0307 -.1257 0.2761 0.6477 -.3920 0.2773 0.6221 0.2253 0.0894

MINPTP2025 0.5349 -.5285 -.0918 -.3371 -.4350 -.1030 -.3971 -.1238 -.6508

MINPT2530 -.4699 0.2824 0.1708 0.1753 0.4048 0.1824 0.2195 -.6619 0.5221

MINPT2030 0.0551 -.2212 0.0732 -.1456 -.0240 0.0737 -.1595 -.7194 -.1126

FPMP2025 0.2974 0.0014 -.1210 0.0707 -.2662 -.1160 0.0007 0.2349 -.1526

FPMP2530 -.1204 -.1543 0.0817 -.0363 0.1239 0.0701 -.0483 0.0027 -.0364

FPMP2030 0.1905 -.1137 -.0559 0.0411 -.1596 -.0602 -.0364 0.2247 -.1689

FETP2025 -.3568 0.1871 0.3687 0.4468 0.3124 0.3677 0.4598 -.4999 0.0292

FEP2530 -.1651 -.0918 -.0200 0.1273 -.1850 0.0019 0.0486 -.0120 0.1851

FEP2030 -.3252 0.0461 0.2030 0.3518 0.0547 0.2177 0.3045 -.3026 0.1464

Correlation Matrix

MINP20 FPM30 FPM25 FPM20 FET30 FET25 FET20 SAP2025 SAP2530

Mb 0.2116 0.1413 0.0048 0.3521 0.2131 0.0803 -.2457 -.0132 -.0579

SA30 -.5575 -.0275 -.1727 -.1621 -.1630 -.3197 -.1183 0.3230 -.3917

SA25 -.1819 -.1214 -.0232 -.1664 -.1970 -.2786 0.0797 -.6331 0.8468

SA20 -.3163 -.0620 -.0854 0.0007 -.2036 -.1127 0.2969 0.3362 0.1851

SP30 -.4578 -.1441 -.0006 -.3148 -.0727 -.3132 -.0012 0.0543 -.1326

SP25 -.1903 -.1148 -.0291 -.1660 -.2189 -.2810 0.0768 -.6265 0.8381

SP20 -.3367 -.0630 -.0988 -.0943 -.1478 -.1337 0.2897 0.3154 0.1978

MINP30 0.1408 -.0045 -.0020 0.2683 0.4549 0.5745 0.0670 0.1857 -.1169

MINP25 0.1821 -.1840 -.1943 -.3613 0.1908 0.4661 0.4409 0.1737 -.1240

MINP20 1.0000 0.0473 0.1163 0.0801 0.2911 0.4923 0.5058 -.0858 0.1308

FPM30 0.0473 1.0000 0.4423 0.1155 -.0520 -.2819 -.3825 0.0774 -.0983

Genome Size Data 11:10 Thursday, October 9, 2008 651

The PRINCOMP Procedure

Correlation Matrix

MINP20 FPM30 FPM25 FPM20 FET30 FET25 FET20 SAP2025 SAP2530

FPM25 0.1163 0.4423 1.0000 0.2804 0.2125 -.0072 -.1705 -.0517 0.0714

FPM20 0.0801 0.1155 0.2804 1.0000 0.0542 0.2290 -.1233 0.1837 -.0678

FET30 0.2911 -.0520 0.2125 0.0542 1.0000 0.4918 0.0196 0.0327 -.0958

FET25 0.4923 -.2819 -.0072 0.2290 0.4918 1.0000 0.5629 0.2046 -.0874

FET20 0.5058 -.3825 -.1705 -.1233 0.0196 0.5629 1.0000 0.1807 0.1380

SAP2025 -.0858 0.0774 -.0517 0.1837 0.0327 0.2046 0.1807 1.0000 -.7639

SAP2530 0.1308 -.0983 0.0714 -.0678 -.0958 -.0874 0.1380 -.7639 1.0000

SAP2030 0.0893 -.0502 0.0424 0.1351 -.1046 0.1320 0.4474 0.1282 0.5421

SPUPP2025 -.1008 0.0677 -.0588 0.0948 0.1036 0.1843 0.1816 0.9733 -.7341

SPUPP2530 0.1309 -.0114 -.0275 0.0564 -.1600 -.0549 0.0744 -.6375 0.8939

SPUPP2030 0.0553 0.0624 -.1029 0.1816 -.0899 0.1390 0.3042 0.2817 0.3250

MINPTP2025 0.6280 0.1821 0.2436 0.3478 0.0738 0.0112 0.0415 -.2037 0.1991

MINPT2530 0.0172 -.1403 -.1506 -.5227 -.2562 -.1470 0.2860 -.0295 0.0071

MINPT2030 0.5864 0.0369 0.0832 -.1633 -.1679 -.1246 0.3000 -.2121 0.1874

FPMP2025 -.0237 -.2619 -.5697 0.6289 -.1197 0.2041 0.0361 0.1969 -.1135

FPMP2530 0.0761 -.4267 0.6221 0.1819 0.2529 0.2372 0.1615 -.1227 0.1592

FPMP2030 0.0341 -.5822 -.0663 0.7402 0.0800 0.3789 0.1588 0.0987 0.0091

FETP2025 0.0759 -.1479 -.1846 -.3680 -.4704 -.3683 0.5612 -.0016 0.2428

FEP2530 0.0975 -.1812 -.2383 0.1341 -.6777 0.3070 0.4539 0.1371 0.0308

FEP2030 0.1127 -.2135 -.2749 -.1230 -.7497 -.0025 0.6470 0.0947 0.1643

Correlation Matrix

SAP2030 SPUPP2025 SPUPP2530 SPUPP2030 MINPTP2025 MINPT2530 MINPT2030

Mb -.1062 -.0481 0.0658 0.0307 0.5349 -.4699 0.0551

SA30 -.1814 0.2900 -.3511 -.1257 -.5285 0.2824 -.2212

SA25 0.4769 -.6202 0.7564 0.2761 -.0918 0.1708 0.0732

SA20 0.7221 0.3223 0.2149 0.6477 -.3371 0.1753 -.1456

SP30 -.1331 0.1147 -.4018 -.3920 -.4350 0.4048 -.0240

SP25 0.4722 -.6194 0.7566 0.2773 -.1030 0.1824 0.0737

SP20 0.7147 0.3529 0.1683 0.6221 -.3971 0.2195 -.1595

MINP30 0.0621 0.1907 0.0058 0.2253 -.1238 -.6619 -.7194

MINP25 0.0356 0.2194 -.1237 0.0894 -.6508 0.5221 -.1126

MINP20 0.0893 -.1008 0.1309 0.0553 0.6280 0.0172 0.5864

FPM30 -.0502 0.0677 -.0114 0.0624 0.1821 -.1403 0.0369

FPM25 0.0424 -.0588 -.0275 -.1029 0.2436 -.1506 0.0832

FPM20 0.1351 0.0948 0.0564 0.1816 0.3478 -.5227 -.1633

FET30 -.1046 0.1036 -.1600 -.0899 0.0738 -.2562 -.1679

FET25 0.1320 0.1843 -.0549 0.1390 0.0112 -.1470 -.1246

FET20 0.4474 0.1816 0.0744 0.3042 0.0415 0.2860 0.3000

SAP2025 0.1282 0.9733 -.6375 0.2817 -.2037 -.0295 -.2121

SAP2530 0.5421 -.7341 0.8939 0.3250 0.1991 0.0071 0.1874

SAP2030 1.0000 0.1392 0.5435 0.8663 0.0407 -.0276 0.0118

SPUPP2025 0.1392 1.0000 -.6721 0.2672 -.2514 0.0019 -.2267

SPUPP2530 0.5435 -.6721 1.0000 0.5340 0.1990 -.1022 0.0871

SPUPP2030 0.8663 0.2672 0.5340 1.0000 -.0281 -.1309 -.1455

Genome Size Data 11:10 Thursday, October 9, 2008 652

The PRINCOMP Procedure

Correlation Matrix

SAP2030 SPUPP2025 SPUPP2530 SPUPP2030 MINPTP2025 MINPT2530 MINPT2030

MINPTP2025 0.0407 -.2514 0.1990 -.0281 1.0000 -.4000 0.5418

MINPT2530 -.0276 0.0019 -.1022 -.1309 -.4000 1.0000 0.5536

MINPT2030 0.0118 -.2267 0.0871 -.1455 0.5418 0.5536 1.0000

FPMP2025 0.0820 0.1274 0.0710 0.2378 0.1025 -.3292 -.2088

FPMP2530 0.0850 -.1208 -.0176 -.1609 0.0876 -.0310 0.0512

FPMP2030 0.1425 0.0324 0.0515 0.1040 0.1600 -.3330 -.1600

FETP2025 0.3710 0.0197 0.1386 0.2029 0.0355 0.4690 0.4623

FEP2530 0.2258 0.0425 0.1284 0.2156 -.0712 0.1559 0.0783

FEP2030 0.3759 0.0412 0.1712 0.2699 -.0288 0.3848 0.3267

Correlation Matrix

FPMP2025 FPMP2530 FPMP2030 FETP2025 FEP2530 FEP2030

Mb 0.2974 -.1204 0.1905 -.3568 -.1651 -.3252

SA30 0.0014 -.1543 -.1137 0.1871 -.0918 0.0461

SA25 -.1210 0.0817 -.0559 0.3687 -.0200 0.2030

SA20 0.0707 -.0363 0.0411 0.4468 0.1273 0.3518

SP30 -.2662 0.1239 -.1596 0.3124 -.1850 0.0547

SP25 -.1160 0.0701 -.0602 0.3677 0.0019 0.2177

SP20 0.0007 -.0483 -.0364 0.4598 0.0486 0.3045

MINP30 0.2349 0.0027 0.2247 -.4999 -.0120 -.3026

MINP25 -.1526 -.0364 -.1689 0.0292 0.1851 0.1464

MINP20 -.0237 0.0761 0.0341 0.0759 0.0975 0.1127

FPM30 -.2619 -.4267 -.5822 -.1479 -.1812 -.2135

FPM25 -.5697 0.6221 -.0663 -.1846 -.2383 -.2749

FPM20 0.6289 0.1819 0.7402 -.3680 0.1341 -.1230

FET30 -.1197 0.2529 0.0800 -.4704 -.6777 -.7497

FET25 0.2041 0.2372 0.3789 -.3683 0.3070 -.0025

FET20 0.0361 0.1615 0.1588 0.5612 0.4539 0.6470

SAP2025 0.1969 -.1227 0.0987 -.0016 0.1371 0.0947

SAP2530 -.1135 0.1592 0.0091 0.2428 0.0308 0.1643

SAP2030 0.0820 0.0850 0.1425 0.3710 0.2258 0.3759

SPUPP2025 0.1274 -.1208 0.0324 0.0197 0.0425 0.0412

SPUPP2530 0.0710 -.0176 0.0515 0.1386 0.1284 0.1712

SPUPP2030 0.2378 -.1609 0.1040 0.2029 0.2156 0.2699

MINPTP2025 0.1025 0.0876 0.1600 0.0355 -.0712 -.0288

MINPT2530 -.3292 -.0310 -.3330 0.4690 0.1559 0.3848

MINPT2030 -.2088 0.0512 -.1600 0.4623 0.0783 0.3267

FPMP2025 1.0000 -.3458 0.6891 -.1637 0.3032 0.1152

FPMP2530 -.3458 1.0000 0.4412 -.0560 -.0761 -.0860

FPMP2030 0.6891 0.4412 1.0000 -.2008 0.2326 0.0442

FETP2025 -.1637 -.0560 -.2008 1.0000 0.2031 0.7302

FEP2530 0.3032 -.0761 0.2326 0.2031 1.0000 0.8173

FEP2030 0.1152 -.0860 0.0442 0.7302 0.8173 1.0000

Genome Size Data 11:10 Thursday, October 9, 2008 653

The PRINCOMP Procedure

Eigenvalues of the Correlation Matrix

Eigenvalue Difference Proportion Cumulative

1 6.50316924 1.42687068 0.2098 0.2098

2 5.07629856 0.64768152 0.1638 0.3735

3 4.42861704 0.98125682 0.1429 0.5164

4 3.44736022 0.67507849 0.1112 0.6276

5 2.77228173 0.39461531 0.0894 0.7170

6 2.37766642 0.24979356 0.0767 0.7937

7 2.12787285 0.73054537 0.0686 0.8624

8 1.39732748 0.50871713 0.0451 0.9074

9 0.88861035 0.08194354 0.0287 0.9361

10 0.80666681 0.40302617 0.0260 0.9621

11 0.40364064 0.10995118 0.0130 0.9751

12 0.29368946 0.00518549 0.0095 0.9846

13 0.28850397 0.13147505 0.0093 0.9939

14 0.15702893 0.13030344 0.0051 0.9990

15 0.02672549 0.02269963 0.0009 0.9999

16 0.00402586 0.00375398 0.0001 1.0000

17 0.00027188 0.00010321 0.0000 1.0000

18 0.00016866 0.00009428 0.0000 1.0000

19 0.00007438 0.00007437 0.0000 1.0000

20 0.00000001 0.00000000 0.0000 1.0000

21 0.00000000 0.00000000 0.0000 1.0000

22 0.00000000 0.00000000 0.0000 1.0000

23 0.00000000 0.00000000 0.0000 1.0000

24 0.00000000 0.00000000 0.0000 1.0000

25 0.00000000 0.00000000 0.0000 1.0000

26 0.00000000 0.00000000 0.0000 1.0000

27 0.00000000 0.00000000 0.0000 1.0000

28 0.00000000 0.00000000 0.0000 1.0000

29 0.00000000 0.00000000 0.0000 1.0000

30 0.00000000 0.00000000 0.0000 1.0000

31 0.00000000 0.0000 1.0000

Genome Size Data 11:10 Thursday, October 9, 2008 654

The PRINCOMP Procedure

Eigenvectors

Prin1 Prin2 Prin3 Prin4 Prin5 Prin6 Prin7 Prin8

Mb -.224875 0.184373 0.060349 -.008957 -.198759 0.082463 -.033955 0.220207

SA30 0.129978 -.311211 -.103060 -.161527 -.071814 -.082513 0.111872 0.098393

SA25 0.314420 0.186420 -.064490 -.181843 0.069049 -.068461 0.009891 0.051539

SA20 0.285617 -.138585 0.170093 -.191539 -.023886 0.159314 0.101265 0.080708

SP30 0.164999 -.242339 -.199492 -.081402 0.077085 -.091047 0.256195 0.102284

SP25 0.317185 0.181918 -.064275 -.179143 0.061035 -.072814 0.004825 0.038484

SP20 0.288546 -.155858 0.141970 -.193441 0.018469 0.174462 0.071053 0.113835

MINP30 -.169837 -.001572 0.245123 -.197261 0.222823 -.036078 -.223054 -.129297

MINP25 0.066772 -.184752 0.090581 0.130348 0.379439 -.084441 -.275136 -.068290

MINP20 -.079574 0.187460 0.124261 0.336897 0.166388 0.171055 -.026361 0.158992

FPM30 -.069158 0.015773 -.128755 -.069957 -.160549 0.440293 -.195885 -.241089

FPM25 -.072601 0.094383 -.101279 -.066473 0.171264 0.340115 0.333731 -.416956

FPM20 -.143406 0.107730 0.238041 -.150115 -.177105 -.017512 0.291241 -.118414

FET30 -.189140 0.023286 0.017779 -.125804 0.402848 0.115564 0.027187 0.382804

FET25 -.119262 0.014448 0.300365 0.113601 0.335151 -.085478 -.033315 -.023846

FET20 0.147608 -.019512 0.263366 0.298957 0.206152 0.015243 0.052385 0.095214

SAP2025 -.087616 -.330239 0.224638 0.026855 -.097516 0.219263 0.080643 0.016267

SAP2530 0.222945 0.341337 -.004573 -.082422 0.103021 -.019341 -.051047 -.004980

SAP2030 0.228530 0.094536 0.285491 -.091696 0.031339 0.255796 0.026563 0.013529

SPUPP2025 -.082007 -.341519 0.200303 0.018391 -.049737 0.239404 0.059843 0.062285

SPUPP2530 0.191042 0.340167 0.074922 -.115965 0.005738 -.007472 -.170695 -.033116

SPUPP2030 0.154980 0.052734 0.326208 -.129914 -.049323 0.263631 -.153808 0.028022

MINPTP2025 -.114294 0.290970 0.024249 0.156959 -.171846 0.198910 0.197412 0.176810

MINPT2530 0.203898 -.143476 -.147673 0.278239 0.098753 -.034042 -.016754 0.061819

MINPT2030 0.083134 0.132803 -.113383 0.397758 -.065582 0.149527 0.164011 0.217336

FPMP2025 -.063897 0.016542 0.286161 -.075876 -.287700 -.290709 -.019427 0.240512

FPMP2530 -.012878 0.082276 0.009976 -.004305 0.311175 -.043523 0.506724 -.216005

FPMP2030 -.071125 0.076815 0.281659 -.074929 -.034621 -.312366 0.372043 0.063003

FETP2025 0.285459 -.036433 -.004549 0.222499 -.103735 0.102737 0.092284 0.130993

FEP2530 0.106010 -.013260 0.234233 0.233444 -.157287 -.198503 -.057856 -.438561

FEP2030 0.241953 -.030690 0.160762 0.293832 -.170787 -.078063 0.013937 -.228921

Eigenvectors

Prin9 Prin10 Prin11 Prin12 Prin13 Prin14 Prin15 Prin16

Mb -.185348 -.179759 0.494084 0.471787 0.404499 0.343763 0.023624 -.006537

SA30 0.278170 0.151196 0.118708 -.239390 0.528483 -.046392 -.012111 -.231925

SA25 0.118173 0.064778 0.077809 0.010785 0.042562 0.047307 0.021178 -.455213

SA20 0.082519 0.006116 0.121571 -.121350 0.104420 0.072029 -.389117 -.203573

SP30 0.147762 0.248423 0.198994 0.208031 -.293125 0.278485 0.087582 0.235585

SP25 0.131683 0.056628 0.093464 0.000360 0.055868 0.051361 0.040050 0.468482

SP20 0.022817 -.003583 0.078990 0.000800 -.106407 0.140191 0.443881 0.226923

MINP30 -.025374 0.373024 -.171810 0.032106 0.111372 0.294089 -.014188 0.004188

MINP25 0.204812 -.198739 -.222943 0.169993 0.143203 0.314158 -.037683 -.002792

MINP20 0.265684 0.169909 -.163746 -.193317 0.080388 0.422062 0.017431 -.000635

FPM30 0.416734 0.091701 0.015763 0.244090 -.042297 -.134352 0.000912 -.017860

Genome Size Data 11:10 Thursday, October 9, 2008 655

The PRINCOMP Procedure

Eigenvectors

Prin9 Prin10 Prin11 Prin12 Prin13 Prin14 Prin15 Prin16

FPM25 0.113804 -.043642 -.020326 0.129900 0.133390 -.030931 0.033698 -.001840

FPM20 0.410288 -.093965 -.196158 0.246734 -.006972 -.069192 0.021588 0.013542

FET30 0.072140 0.009005 0.049745 0.075778 -.017271 -.289616 -.025457 0.011837

FET25 0.189791 0.036363 0.453848 -.053916 -.087403 -.266501 0.043895 -.007542

FET20 -.021281 0.266399 0.094318 0.261300 0.100261 -.357009 0.015719 0.005933

SAP2025 -.055370 -.065704 0.024310 -.121530 0.047568 0.013077 -.374956 0.316584

SAP2530 -.039734 -.021088 0.008554 0.139002 -.245022 0.069069 0.026257 -.299214

SAP2030 -.133165 -.117966 0.044801 0.055364 -.314608 0.123173 -.447909 -.047582

SPUPP2025 -.122915 -.065132 -.029695 0.000339 -.158530 0.072374 0.363028 -.303725

SPUPP2530 0.025074 -.115735 -.046610 -.142014 0.254129 -.141354 -.021554 0.287726

SPUPP2030 -.107715 -.224982 -.094564 -.184425 0.149704 -.101316 0.386459 0.027641

MINPTP2025 0.043036 0.288474 0.049994 -.283814 -.051274 0.077239 0.043227 0.001611

MINPT2530 0.183272 -.488633 -.021542 0.104647 0.012964 -.016128 -.016767 -.005544

MINPT2030 0.207158 -.186007 0.025697 -.161896 -.034727 0.055482 0.023838 -.003872

FPMP2025 0.258374 -.037385 -.152325 0.111672 -.113163 -.037063 0.000780 0.019963

FPMP2530 -.251348 -.120307 -.038422 -.080823 0.163121 0.092975 0.052431 0.015389

FPMP2030 0.053004 -.137030 -.175585 0.039332 0.027469 0.035491 0.005631 -.034738

FETP2025 -.213951 0.263275 -.348310 0.347984 0.200238 -.134668 -.026563 0.013743

FEP2530 0.081389 0.020880 0.328845 -.128391 -.054935 0.091544 0.064636 -.018689

FEP2030 -.069085 0.169480 0.024483 0.115131 0.079532 -.015453 0.029815 -.005190

Eigenvectors

Prin17 Prin18 Prin19 Prin20 Prin21 Prin22 Prin23 Prin24

Mb 0.001202 -.001520 0.000662 -.000005 0.000007 0.000036 0.000034 0.000013

SA30 0.012588 -.003625 -.006193 -.000065 0.000081 0.000069 -.000045 -.000003

SA25 0.015681 -.002722 -.013648 -.000103 0.000145 0.000184 -.000031 -.000003

SA20 0.017452 -.003103 -.005541 -.000083 -.000031 0.000130 0.000023 -.000017

SP30 -.014518 0.001557 0.006759 0.000036 -.000062 -.000064 0.000042 0.000016

SP25 -.018368 0.001753 0.014886 0.000098 -.000124 -.000190 0.000031 0.000013

SP20 -.018995 0.002791 0.008047 0.000090 0.000032 -.000124 -.000020 0.000033

MINP30 -.001409 -.000031 -.002126 0.170704 -.030209 0.377794 -.050584 0.015465

MINP25 0.002282 0.000026 -.002713 -.210888 0.026190 -.472061 0.140515 0.369201

MINP20 -.000733 0.000851 -.000347 0.059386 0.000300 0.136625 -.093679 -.373439

FPM30 0.108259 -.211847 0.581982 -.000050 -.000143 -.000790 -.001113 0.000836

FPM25 -.041228 0.672864 -.185546 0.000891 -.000612 -.000407 0.000901 -.000136

FPM20 -.091639 -.452412 -.512864 -.000897 0.000864 0.001400 0.000419 -.000880

FET30 0.000836 -.003119 0.002951 0.081533 0.615920 0.063455 0.364827 -.054824

FET25 -.000729 -.002305 0.000628 0.480124 -.320501 -.266480 -.171842 0.021701

FET20 -.002351 -.002147 0.000450 -.610136 -.174458 0.244202 -.123630 0.023210

SAP2025 -.001471 0.000189 0.009999 0.000038 -.000187 -.000085 0.000055 -.000011

SAP2530 0.007830 -.000583 -.009380 -.000061 0.000091 0.000134 -.000004 -.000001

SAP2030 0.010118 -.000651 -.001395 -.000044 -.000104 0.000096 0.000065 -.000017

SPUPP2025 0.002654 0.000643 -.008885 -.000025 0.000165 0.000094 -.000053 0.000017

SPUPP2530 -.007667 0.000615 0.009640 0.000069 -.000076 -.000138 0.000001 0.000001

SPUPP2030 -.006947 0.001534 0.002401 0.000062 0.000089 -.000073 -.000058 0.000020

Genome Size Data 11:10 Thursday, October 9, 2008 656

The PRINCOMP Procedure

Eigenvectors

Prin17 Prin18 Prin19 Prin20 Prin21 Prin22 Prin23 Prin24

MINPTP2025 -.002196 -.000156 -.000348 0.012164 -.023031 0.016817 0.143416 0.733212

MINPT2530 0.004263 0.000700 0.001246 0.281200 -.056667 0.618980 -.034423 0.269203

MINPT2030 0.000638 0.000623 0.001497 -.098007 0.024928 -.213261 -.024334 -.274649

FPMP2025 0.622753 0.412854 0.076244 0.000101 -.000700 -.001050 -.000564 0.000079

FPMP2530 0.543731 -.335679 0.242018 -.000763 0.000068 -.000444 -.001352 0.000199

FPMP2030 -.541089 0.120317 0.546320 0.000982 -.000306 -.000609 0.000070 0.000982

FETP2025 -.001587 -.002792 -.002937 0.421955 0.224572 -.222281 -.347545 0.073130

FEP2530 -.000687 0.003887 -.000409 -.068213 0.645877 0.051979 -.208648 0.061059

FEP2030 -.000100 -.000551 -.000371 0.204690 -.118035 0.008728 0.777392 -.159370

Eigenvectors

Prin25 Prin26 Prin27 Prin28 Prin29 Prin30 Prin31

Mb -.000000 0.000000 0.000000 0.000000 0.000000 -.000000 0.000000

SA30 0.232374 -.000000 0.357736 0.000000 -.000000 -.355212 0.000000

SA25 -.354204 -.000000 0.278383 0.000000 0.000000 0.614416 0.000000

SA20 -.039234 -.000000 -.737358 0.000000 0.000000 0.000000 0.000000

SP30 -.000000 0.277730 -.000000 0.369255 0.000000 -.000000 -.412893

SP25 0.000000 -.351247 -.000000 0.324308 -.000000 0.000000 0.578196

SP20 -.000000 -.031610 0.000000 -.705963 0.000000 0.000000 0.000000

MINP30 -.000000 0.000000 0.000000 0.000000 0.556504 -.000000 0.000000

MINP25 -.000000 0.000000 -.000000 -.000000 0.000000 -.000000 -.000000

MINP20 0.000000 -.000000 0.000000 0.000000 -.477176 0.000000 -.000000

FPM30 -.000000 0.000000 -.000000 -.000000 0.000000 -.000000 0.000000

FPM25 0.000000 0.000000 0.000000 -.000000 -.000000 0.000000 -.000000

FPM20 0.000000 -.000000 0.000000 0.000000 -.000000 0.000000 0.000000

FET30 0.000000 0.000000 0.000000 0.000000 -.000000 0.000000 -.000000

FET25 -.000000 0.000000 0.000000 -.000000 -.000000 -.000000 -.000000

FET20 -.000000 -.000000 -.000000 0.000000 0.000000 -.000000 0.000000

SAP2025 0.354035 0.000000 0.253161 -.000000 0.000000 0.558749 -.000000

SAP2530 0.797972 0.000000 0.000000 0.000000 0.000000 0.000000 0.000000

SAP2030 -.238540 -.000000 0.432136 -.000000 -.000000 -.429087 0.000000

SPUPP2025 0.000000 0.354666 -.000000 0.296930 -.000000 0.000000 0.529386

SPUPP2530 -.000000 0.770763 0.000000 0.000000 0.000000 0.000000 0.000000

SPUPP2030 -.000000 -.280408 -.000000 0.414637 0.000000 -.000000 -.463638

MINPTP2025 -.000000 0.000000 -.000000 -.000000 0.000000 -.000000 0.000000

MINPT2530 -.000000 0.000000 0.000000 0.000000 0.000000 -.000000 0.000000

MINPT2030 0.000000 -.000000 0.000000 0.000000 0.680152 0.000000 0.000000

FPMP2025 -.000000 0.000000 -.000000 -.000000 -.000000 -.000000 0.000000

FPMP2530 -.000000 -.000000 -.000000 -.000000 0.000000 -.000000 0.000000

FPMP2030 -.000000 0.000000 -.000000 -.000000 0.000000 -.000000 -.000000

FETP2025 -.000000 0.000000 -.000000 0.000000 0.000000 -.000000 0.000000

FEP2530 -.000000 0.000000 -.000000 0.000000 0.000000 -.000000 0.000000

FEP2030 0.000000 0.000000 0.000000 -.000000 -.000000 0.000000 -.000000

Genome Size Data 11:10 Thursday, October 9, 2008 657

Plot of Prin1*Prin2. Symbol is value of GenomeSize. Capital letters are large genomes, lower case letters are small genomes.

Prin1 ‚

‚

‚

‚

‚

‚

‚

5 ˆ

‚

‚

‚

‚ Q

4 ˆ

‚ sx

‚

‚ m

‚ p E

3 ˆ v

‚ y

‚ A

‚

‚ i g

2 ˆ uz k

‚ a

‚

‚ w F

‚ b

1 ˆ

‚ T

‚ d

‚ R M

‚ r

0 ˆ q

‚ h

‚ e

‚ W P

‚ N

-1 ˆ o

‚ G

‚

‚

‚ B

-2 ˆ

‚ t H

‚ S U C

‚ Y

‚

-3 ˆ K

‚

‚

‚ J

‚ ZD L

-4 ˆ

‚

‚

‚

‚ V

-5 ˆ X

‚

Šƒƒˆƒƒƒƒƒƒƒƒƒƒƒƒˆƒƒƒƒƒƒƒƒƒƒƒƒˆƒƒƒƒƒƒƒƒƒƒƒƒˆƒƒƒƒƒƒƒƒƒƒƒƒˆƒƒƒƒƒƒƒƒƒƒƒƒˆƒƒƒƒƒƒƒƒƒƒƒƒˆƒƒ

-6 -4 -2 0 2 4 6

Prin2

NOTE: 5 obs had missing values.

Genome Size Data 11:10 Thursday, October 9, 2008 659

Plot of Prin1*Prin3. Symbol is value of GenomeSize.

Prin1 ‚

‚

‚

‚

‚

‚

‚

5 ˆ

‚

‚

‚

‚ Q

4 ˆ

‚ x s

‚

‚ m

‚ p E

3 ˆ v

‚ y

‚ A

‚

‚ g i

2 ˆ u k z

‚ a

‚

‚ F w

‚ b

1 ˆ

‚ T

‚ d

‚ M R

‚ r

0 ˆ q

‚ h

‚ e

‚ W P

‚ N

-1 ˆ o

‚ G

‚

‚

‚ B

-2 ˆ

‚ H

‚ U S C

‚ Y

‚

-3 ˆ K

‚

‚

‚ J

‚ L D Z

-4 ˆ

‚

‚

‚

‚ V

-5 ˆ X

‚

Šƒƒˆƒƒƒƒƒƒƒƒƒƒƒƒˆƒƒƒƒƒƒƒƒƒƒƒƒˆƒƒƒƒƒƒƒƒƒƒƒƒˆƒƒƒƒƒƒƒƒƒƒƒƒˆƒƒƒƒƒƒƒƒƒƒƒƒˆƒƒƒƒƒƒƒƒƒƒƒƒˆƒƒ

-6 -4 -2 0 2 4 6

Prin3

NOTE: 5 obs had missing values. 1 obs hidden.

Genome Size Data 11:10 Thursday, October 9, 2008 661

Plot of Prin1*Prin4. Symbol is value of GenomeSize.

Prin1 ‚

‚

‚

‚

‚

‚

‚

5 ˆ

‚

‚

‚

‚ Q

4 ˆ

‚ x s

‚

‚ m

‚ E p

3 ˆ v

‚ y

‚ A

‚

‚ g i

2 ˆ z k u

‚ a

‚

‚ F w

‚ b

1 ˆ

‚ T

‚ d

‚ R M

‚ r

0 ˆ q

‚ h

‚ e

‚ P W

‚ N

-1 ˆ o

‚ G

‚

‚

‚ B

-2 ˆ

‚ t H

‚ U S C

‚ Y

‚

-3 ˆ K

‚

‚

‚ J

‚ D Z L

-4 ˆ

‚

‚

‚

‚ V

-5 ˆ X

‚

Šˆƒƒƒƒƒƒƒƒƒˆƒƒƒƒƒƒƒƒƒˆƒƒƒƒƒƒƒƒƒˆƒƒƒƒƒƒƒƒƒˆƒƒƒƒƒƒƒƒƒˆƒƒƒƒƒƒƒƒƒˆƒƒƒƒƒƒƒƒƒˆƒƒƒƒƒƒƒƒƒˆƒƒƒƒƒƒƒƒƒˆ

-5 -4 -3 -2 -1 0 1 2 3 4

Prin4

NOTE: 5 obs had missing values.

Genome Size Data 11:10 Thursday, October 9, 2008 663

Plot of Prin1*Prin5. Symbol is value of GenomeSize.

Prin1 ‚

‚

‚

‚

‚

‚

‚

5 ˆ

‚

‚

‚

‚ Q

4 ˆ

‚ x s

‚

‚ m

‚ p E

3 ˆ v

‚ y

‚ A

‚

‚ i g

2 ˆ zu k

‚ a

‚

‚ F w

‚ b

1 ˆ

‚ T

‚ d

‚ R M

‚ r

0 ˆ q

‚ h

‚ e

‚ P W

‚ N

-1 ˆ o

‚ G

‚

‚

‚ B

-2 ˆ

‚ tH

‚ S C U

‚ Y

‚

-3 ˆ K

‚

‚

‚ J

‚ Z L D

-4 ˆ

‚

‚

‚

‚ V

-5 ˆ X

‚

Šˆƒƒƒƒƒƒƒƒˆƒƒƒƒƒƒƒƒˆƒƒƒƒƒƒƒƒˆƒƒƒƒƒƒƒƒˆƒƒƒƒƒƒƒƒˆƒƒƒƒƒƒƒƒˆƒƒƒƒƒƒƒƒˆƒƒƒƒƒƒƒƒˆƒƒƒƒƒƒƒƒˆƒƒƒƒƒƒƒƒˆ

-5 -4 -3 -2 -1 0 1 2 3 4 5

Prin5

NOTE: 5 obs had missing values.

Genome Size Data 11:10 Thursday, October 9, 2008 665

Plot of Prin2*Prin3. Symbol is value of GenomeSize.

Prin2 ‚

‚

6 ˆ

‚

‚

‚

‚ M

5 ˆ

‚

‚

‚

‚

4 ˆ

‚ Q E

‚

‚

‚

3 ˆ

‚

‚ x k

‚ v s

‚ G

2 ˆ g H

‚ L N P

‚ C

‚ J F Y

‚ e

1 ˆ

‚

‚ D

‚ V Z

‚

0 ˆ U

‚ B X

‚ K R

‚

‚ i y

-1 ˆ m

‚

‚ b

‚ q

‚ T z r

-2 ˆ u S

‚ h w

‚ o

‚ A

‚ t W

-3 ˆ

‚ d

‚

‚

‚ p

-4 ˆ

‚

‚ a

‚

‚

-5 ˆ

‚

Šƒƒˆƒƒƒƒƒƒƒƒƒƒƒƒˆƒƒƒƒƒƒƒƒƒƒƒƒˆƒƒƒƒƒƒƒƒƒƒƒƒˆƒƒƒƒƒƒƒƒƒƒƒƒˆƒƒƒƒƒƒƒƒƒƒƒƒˆƒƒƒƒƒƒƒƒƒƒƒƒˆƒƒ

-6 -4 -2 0 2 4 6

Prin3

NOTE: 5 obs had missing values.

Genome Size Data 11:10 Thursday, October 9, 2008 667

Plot of Prin2*Prin4. Symbol is value of GenomeSize.

Prin2 ‚

‚

6 ˆ

‚

‚

‚

‚ M

5 ˆ

‚

‚

‚

‚

4 ˆ

‚ E Q

‚

‚

‚

3 ˆ

‚

‚ x k

‚ v s

‚ G

2 ˆ g H

‚ N L P

‚ C

‚ F Y J

‚ e

1 ˆ

‚

‚ D

‚ V Z

‚

0 ˆ U

‚ X B

‚ K R

‚

‚ y i

-1 ˆ m

‚

‚ b

‚ q

‚ r z T

-2 ˆ S u

‚ h w

‚ o

‚ A

‚ t W

-3 ˆ

‚ d

‚

‚

‚ p

-4 ˆ

‚

‚ a

‚

‚

-5 ˆ

‚

Šˆƒƒƒƒƒƒƒƒƒˆƒƒƒƒƒƒƒƒƒˆƒƒƒƒƒƒƒƒƒˆƒƒƒƒƒƒƒƒƒˆƒƒƒƒƒƒƒƒƒˆƒƒƒƒƒƒƒƒƒˆƒƒƒƒƒƒƒƒƒˆƒƒƒƒƒƒƒƒƒˆƒƒƒƒƒƒƒƒƒˆ

-5 -4 -3 -2 -1 0 1 2 3 4

Prin4

NOTE: 5 obs had missing values.

Genome Size Data 11:10 Thursday, October 9, 2008 669

Plot of Prin2*Prin5. Symbol is value of GenomeSize.

Prin2 ‚

‚

6 ˆ

‚

‚

‚

‚ M

5 ˆ

‚

‚

‚

‚

4 ˆ

‚ Q E

‚

‚

‚

3 ˆ

‚

‚ x k

‚ v s

‚ G

2 ˆ H g

‚ P LN

‚ C

‚ J Y F

‚ e

1 ˆ

‚

‚ D

‚ Z V

‚

0 ˆ U

‚ B X

‚ R K

‚

‚ y i

-1 ˆ m

‚

‚ b

‚ q

‚ Tz r

-2 ˆ S u

‚ h w

‚ o

‚ A

‚ t W

-3 ˆ

‚ d

‚

‚

‚ p

-4 ˆ

‚

‚ a

‚

‚

-5 ˆ

‚

Šˆƒƒƒƒƒƒƒƒˆƒƒƒƒƒƒƒƒˆƒƒƒƒƒƒƒƒˆƒƒƒƒƒƒƒƒˆƒƒƒƒƒƒƒƒˆƒƒƒƒƒƒƒƒˆƒƒƒƒƒƒƒƒˆƒƒƒƒƒƒƒƒˆƒƒƒƒƒƒƒƒˆƒƒƒƒƒƒƒƒˆ

-5 -4 -3 -2 -1 0 1 2 3 4 5

Prin5

NOTE: 5 obs had missing values.

Genome Size Data 11:10 Thursday, October 9, 2008 671

Plot of Prin3*Prin4. Symbol is value of GenomeSize.

Prin3 ‚

‚

‚

‚

‚

‚

‚

5 ˆ

‚

‚ o

‚

‚

4 ˆ C

‚

‚ E

‚

‚

3 ˆ P

‚ Q

‚

‚ Z

‚

2 ˆ S w

‚

‚ p r

‚ z

‚ R

1 ˆ Y W

‚ A

‚ F a y s

‚ K

‚ b k

0 ˆ D N T

‚ t H e

‚ X q

‚

‚

-1 ˆ U B i

‚

‚ x

‚ L M

‚ V J

-2 ˆ

‚

‚ g

‚

‚ v

-3 ˆ m

‚

‚ h

‚ G

‚

-4 ˆ d

‚

‚

‚ u

‚

-5 ˆ

‚

Šˆƒƒƒƒƒƒƒƒƒˆƒƒƒƒƒƒƒƒƒˆƒƒƒƒƒƒƒƒƒˆƒƒƒƒƒƒƒƒƒˆƒƒƒƒƒƒƒƒƒˆƒƒƒƒƒƒƒƒƒˆƒƒƒƒƒƒƒƒƒˆƒƒƒƒƒƒƒƒƒˆƒƒƒƒƒƒƒƒƒˆ

-5 -4 -3 -2 -1 0 1 2 3 4

Prin4

NOTE: 5 obs had missing values.

Genome Size Data 11:10 Thursday, October 9, 2008 673

Plot of Prin3*Prin5. Symbol is value of GenomeSize.

Prin3 ‚

‚

‚

‚

‚

‚

‚

5 ˆ

‚

‚ o

‚

‚

4 ˆ C

‚

‚ E

‚

‚

3 ˆ P

‚ Q

‚

‚ Z

‚

2 ˆ S w

‚

‚ p r

‚ z

‚ R

1 ˆ Y W

‚ A

‚ y F a s

‚ K

‚ b k

0 ˆ N D T

‚ tH e

‚ q X

‚

‚

-1 ˆ B U i

‚

‚ x

‚ L M

‚ J V

-2 ˆ

‚

‚ g

‚

‚ v

-3 ˆ m

‚

‚ h

‚ G

‚

-4 ˆ d

‚

‚

‚ u

‚

-5 ˆ

‚

Šˆƒƒƒƒƒƒƒƒˆƒƒƒƒƒƒƒƒˆƒƒƒƒƒƒƒƒˆƒƒƒƒƒƒƒƒˆƒƒƒƒƒƒƒƒˆƒƒƒƒƒƒƒƒˆƒƒƒƒƒƒƒƒˆƒƒƒƒƒƒƒƒˆƒƒƒƒƒƒƒƒˆƒƒƒƒƒƒƒƒˆ

-5 -4 -3 -2 -1 0 1 2 3 4 5

Prin5

NOTE: 5 obs had missing values.

Genome Size Data 11:10 Thursday, October 9, 2008 675

Plot of Prin4*Prin5. Symbol is value of GenomeSize.

Prin4 ‚

‚

‚

4 ˆ

‚ T

‚

‚

‚

‚

3 ˆ

‚ M u

‚ e

‚

‚ R

‚ q

2 ˆ G

‚

‚ W s k

‚ z

‚

‚ H r i

1 ˆ P x

‚

‚ p b

‚ w B

‚ J L

‚ Y

0 ˆ o

‚ C Z

‚ A N K

‚

‚ Q

‚ d

-1 ˆ S D

‚ U

‚

‚

‚

‚ y t

-2 ˆ X V

‚ h

‚ g

‚ a

‚ F

‚ m

-3 ˆ

‚

‚ v

‚

‚

‚

-4 ˆ

‚

‚ E

‚

‚

‚

-5 ˆ

‚

Šˆƒƒƒƒƒƒƒƒˆƒƒƒƒƒƒƒƒˆƒƒƒƒƒƒƒƒˆƒƒƒƒƒƒƒƒˆƒƒƒƒƒƒƒƒˆƒƒƒƒƒƒƒƒˆƒƒƒƒƒƒƒƒˆƒƒƒƒƒƒƒƒˆƒƒƒƒƒƒƒƒˆƒƒƒƒƒƒƒƒˆ

-5 -4 -3 -2 -1 0 1 2 3 4 5

Prin5

NOTE: 5 obs had missing values.
