# Supplementary material for: Intrapopulation Genome Size Variation in D. melanogaster Reflects Life History Variation and Plasticity
Source: PLoS Genet. 2014 Jul 24;10(7):e1004522. doi: 10.1371/journal.pgen.1004522 (PMC4109859; doi:10.1371/journal.pgen.1004522)
Supplement: Table S14 — Drosophila diet composition. (DOCX) [file pgen.1004522.s017.docx]

**Table S14: Drosophila diet composition**.

|  | **% Macronutrient Content** | | |  |
| --- | --- | --- | --- | --- |
| **Ingredient** | **Protein** | **Soluble CHO*** | **Lipids** | **Amount**** |
| Cornmeal | 9.9 | 63.5 | 5.9 | 95.5 g |
| Light corn syrup | 0 | 76.8 | 0.2 | 68.3 mL |
| Malt extract | 13.5 | 68.2 | 9.1 | 67 g |
| Yeast | 45 | 24 | 2 | 27.3 g |
| Soy flour | 34.5 | 25.6 | 20.7 | 22.8 g |
| Diet Total | 13.7 | 60.9 | 6.1 | 1000 mL |

*CHO = Cholesterol; ** g = grams, mL = milliliters
